# Severity of surgical histopathological fibrosis predicted postoperative recurrence in Crohn’s disease: a multi-center retrospective cohort study

**DOI:** 10.1007/s00535-026-02374-9

**Published:** 2026-02-26

**Authors:** Xinyu Wang, Yiwen Tu, Shuowen Zhang, Tianyi Che, Shenglan You, Weitong Gao, Lingying Zhao, Ren Mao, Jing Sun, Yubei Gu, Yao Zhang, Zirui He, Yi Li, Duowu Zou

**Affiliations:** 1 Department of Gastroenterology, Ruijin Hospital, Shanghai Jiao Tong University School of Medicine, 197 Ruijin 2nd Road, Shanghai, 200025 China; 2 Department of Gastroenterology, The First Affiliated Hospital, Sun Yat-Sen University School of Medicine, Guangzhou, China; 3 Department of General Surgery, Ruijin Hospital, Shanghai Jiao Tong University School of Medicine, 197 Ruijin 2nd Road, Shanghai, 200025 China; 4 Department of General Surgery, Jinling Hospital, Nanjing University School of Medicine, No. 305 East Zhongshan Road, Nanjing, 210002 Jiangsu Province China

**Keywords:** Crohn’s disease, Fibrosis, Postoperative recurrence

## Abstract

**Background:**

Despite treatment advances, intestinal surgery remains common in Crohn’s disease (CD), with over half of patients experiencing postoperative recurrence. Intestinal fibrosis represents a key pathological feature underlying this clinical course. This study aimed to investigate the relationship between fibrosis severity and the risk of postoperative recurrence.

**Methods:**

A multi-center retrospective cohort study included CD patients undergoing intestinal resection. Histopathological slides from lesion sites and resection margins were analyzed using Masson’s trichrome staining to quantify the proportion of collagen fiber area, representing fibrosis extent. Postoperative endoscopic and clinical recurrence data were collected via electronic medical records and patient follow-up interviews. Multivariable Cox regression models estimated hazard ratios (HRs) with 95% confidence intervals (CIs) for the associations between fibrosis severity and recurrence risk.

**Results:**

Among 268 patients, endoscopic recurrence occurred in 84 (31.3%) and clinical recurrence in 91 (34.0%). The degree of transmural intestinal fibrosis was positively associated with postoperative endoscopic recurrence (lesion site: HR_per SD_ = 1.46, 95% CI 1.18–1.80; resection margin: HR_per SD_ = 1.35, 95% CI 1.12–1.63) and clinical recurrence (lesion site: HR_per SD_ = 1.95, 95% CI 1.59–2.39; resection margin: HR_per SD_ = 1.29, 95% CI 1.09–1.54). Significant associations persisted when analyzing fibrosis in the mucosal, submucosal, and muscularis propria layers individually.

**Conclusion:**

The severity of intestinal fibrosis in both lesion site and resection margin independently predicted an increased risk of postoperative endoscopic and clinical recurrence in CD. Histopathological fibrosis assessment may help identify high-risk individuals prone to postoperative recurrence, potentially informing personalized postoperative management strategies.

**Supplementary Information:**

The online version contains supplementary material available at 10.1007/s00535-026-02374-9.

## Introduction

Crohn’s disease (CD), a major form of inflammatory bowel disease (IBD), exhibits rising global incidence with prevalence rates of approximately 322 per 100,000 persons in Europe, 319 per 100,000 in North America, and 3.39 per 100,000 in China [1–3]. The disease frequently manifests with symptoms such as diarrhea, abdominal pain, and weight loss, often progressing to complications such as strictures, fistulae, and abscesses [4]. Consequently, bowel resection is frequently required for CD patients, as epidemiological studies have demonstrated that over 70% of patients required surgery within 10 years of diagnosis due to complications such as strictures, perforations, or uncontrolled inflammation [5]. Despite surgical intervention, recurrence remains a major challenge. Endoscopic recurrence occurs in 35–85% of patients within the first postoperative year, clinical recurrence in 10–38%, and 18% require reoperation within 5 years [6, 7]. This high recurrence burden underscores the urgent need for improved strategies to predict and prevent postoperative recurrence.

Identifying robust risk factors is paramount for developing precise risk stratification and management strategies aimed at reducing postoperative recurrence. Established risk factors such as smoking, previous intestinal surgeries, penetrating disease behaviors, and perianal disease have been recognized and included in models guiding postoperative prophylactic therapy [8]. However, the predictive value of pathological indicators within resected intestinal tissue for the recurrence of CD remains a matter of debate. While features such as granulomas, active inflammation, and plexitis have been studied, their predictive performance for recurrence is limited [9], highlighting the need for novel, reliable pathological predictors.

Intestinal fibrosis, leading to stricture formation, is a primary driver for surgical intervention in CD [10, 11]. In addition, intestinal fibrosis represents one of the most prevalent pathological features in resected specimens [12]. Despite its clinical significance and ubiquitous presence in surgical CD, the relationship between a quantitatively assessed degree of intestinal fibrosis and the risk of postoperative recurrence has not been systematically investigated. Understanding this link could provide crucial prognostic information.

Therefore, the objective of this study is to establish a multi-center cohort to quantitatively assess the degree of intestinal fibrosis and to explore its association with the risk of postoperative endoscopic recurrence and clinical recurrence in CD patients.

## Methods

### Study design and population

**Fig. 1:**
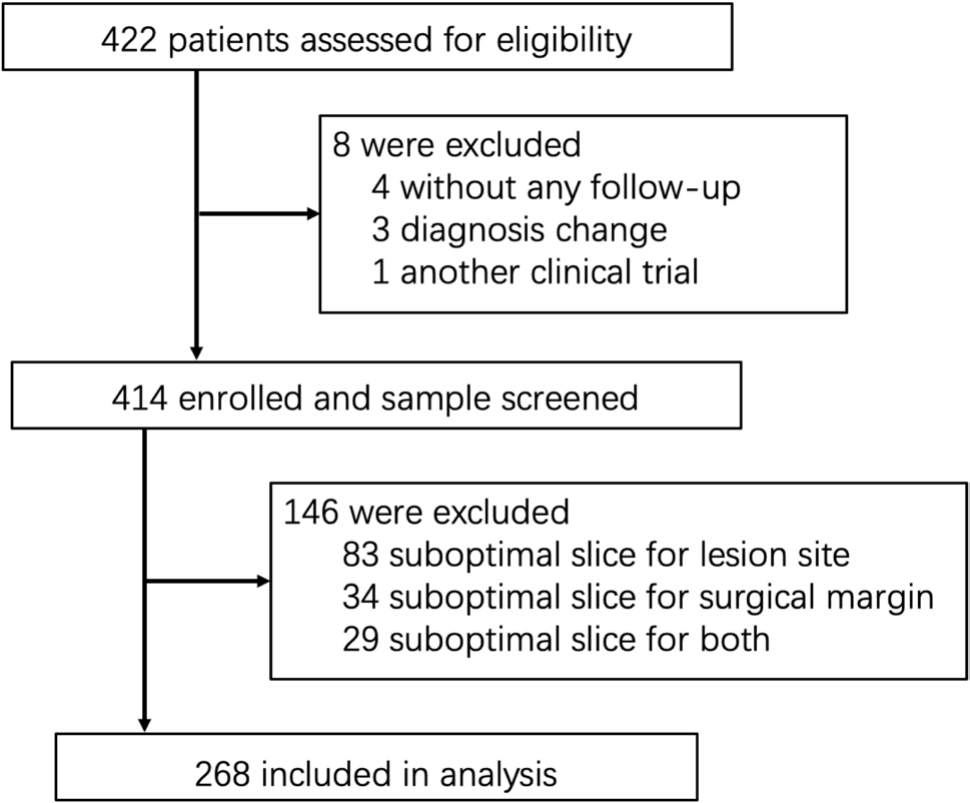
Flowchart of patient selection for cohort

We conducted a multi-center, retrospective observational study in 3 medical centers, including the Ruijin Hospital affiliated with Shanghai Jiao Tong University School of Medicine, Jinling Hospital affiliated with Nanjing University School of Medicine, and the First Affiliated Hospital of Sun Yat-Sen University School of Medicine. A total of 422 CD patients who had undergone surgery from January 2010 to December 2023 at one of the three medical centers were enrolled in this study. The diagnosis of CD was based on diagnostic criteria of the European Crohn’s Disease and Colitis Organization (ECCO) and was confirmed by at least two gastroenterologists from tertiary hospitals through clinical feature manifestations, laboratory test indicators, endoscopic examination, and imaging examination. The diagnosis and treatment process of the patients are completely recorded in the electronic medical record system. We performed a rigorous quality assessment of all available tissue sections. Slides were excluded primarily due to compromised histological integrity, as evidenced by the absence of the mucosal or muscularis propria, or due to significant preparation artifacts (Fig. S1A–C). This process resulted in the exclusion of 146 cases from the initial cohort. After excluding patients whose tissue samples did not contain all layers of the intestinal wall, those who lost follow-up, those with diagnostic changes, and those who participated in another clinical trial, the final study cohort included 268 cases with the highest quality tissue samples constituted our study population for detailed pathological analysis (Figs. 1 and S1D). This study adhered to STROBE guidelines for reporting observational research. The completed checklist is provided in Supplementary file 1.

### Tissue procurement and pathological evaluation

The extent of intestinal resection was determined intraoperatively based on macroscopic assessment, including bowel wall thickness, luminal narrowing, proximal dilatation, and tissue pliability. In accordance with the clinical practice guidelines of the American Society of Colon and Rectal Surgeons [13], the surgical aim was to resect the grossly affected bowel segment together with a margin of approximately 2 cm of macroscopically normal appearing intestine on both the proximal and distal sides. This approach sought to balance the completeness of macroscopic resection with the preservation of intestinal length, thereby mitigating long-term risks such as short bowel syndrome and malnutrition. The final macroscopic margin length was confirmed by pathological measurement of the fresh specimen.

Resected specimens underwent gross anatomical examination by experienced gastroenterologists to identify regions of interest. This identification was based on morphological features including bowel wall thickness, luminal narrowing, proximal dilatation, and tissue pliability [14]. Subsequently, a dedicated pathologist evaluated the specimen and dissected tissue blocks specifically from the central or most severe region of each primary lesion, such as the narrowest point of a stricture or the tract of a fistula, for histological analysis. Formalin-fixed, paraffin-embedded sections were stained with hematoxylin and eosin (H&E) and evaluated for active inflammation according to standard criteria, which comprised neutrophil infiltration, cryptitis, crypt architectural distortion, and ulceration [15]. In addition, Masson’s trichrome staining was performed on tissue sections to evaluate collagen deposition and the extent of fibrosis.

### Ascertainment of intestinal fibrosis

The primary variable of interest for this study was the degree of transmural intestinal fibrosis at the lesion site and the resection margin site (calculated based on the proportion of the blue area in Masson-stained pathological sections, classified as continuous variables and trichotomized categorical variables). In this study, pathological sections from two sites of each sample, the intestinal lesion site (narrowing, obstruction, or significantly thickened intestinal wall area) and the resection margin (the margin on the same side as the lesion site), were analyzed. The degree of intestinal fibrosis was quantified by software-identified collagen area fraction in Masson-stained tissue sections (total collagen fiber area/intestinal tissue specimen area × 100%, the methodology is described in detail in the supplementary materials) [16]. Collagen area fraction was analyzed both as a continuous variable (per standard deviation increase) and as a trichotomized categorical variable (stratified into high, moderate, and low groups).

Moreover, granuloma was defined as the aggregation of epithelioid tissue cells (such as monocytes, macrophages), which may contain multinucleated giant cells. Plexitis was defined as the presence of more than three inflammatory cells (such as neutrophils, lymphocytes, plasma cells, eosinophils) around the submucosal or myenteric plexus. Pathological scoring was accomplished by two researchers. For occasional controversial samples, the pathological score was determined after discussion and consensus by experienced CD pathologists. Considering that most pathological specimens did not include the complete serosa, all serosal layer data were excluded in the analysis of the study results.

### Data collection and study endpoints

Postoperative follow-up adhered to the routine clinical practice at each center. Typically, the first outpatient visit was scheduled 1 month after surgery, with subsequent visits arranged at 3 to 6 months’ intervals. The first endoscopic examination was generally performed 6 months postoperatively, followed by annual surveillance. Earlier endoscopic evaluation was performed if clinically indicated. The postoperative follow-up duration for the entire cohort was calculated from the date of surgery to the date of the last documented clinical or endoscopic visit. The median follow-up time was 43.5 months (interquartile range, 23.4–69.5 months); the mean follow-up was 50.1 months (Fig. S2). Researchers established an electronic clinical database using standardized protocols. Each patient record was uniquely numbered to ensure traceability. To guarantee data accuracy, two independent investigators collected clinical information and conducted follow-up using a unified form. Clinical outcome follow-up was conducted through a combination of electronic medical record system review and telephone follow-up, with multiple records and methods cross-verified to minimize bias. Electronic medical record review captured surgical records, treatment plans, endoscopic reports (gastroscopy/colonoscopy), imaging data (computed tomography/magnetic resonance imaging/intestinal ultrasound), and laboratory test indicators (albumin/C-reactive protein), while telephone follow-up documented symptoms, postoperative medication regimens, and external hospital records. The disease activity was judged by experienced experts in the diagnosis and treatment of CD.

The primary outcomes included Endoscopic Recurrence and Clinical Recurrence. Endoscopic recurrence was defined by anastomotic recurrence (modified Rutgeerts score ≥ i2b) [17] and non-anastomotic recurrence (SES-CD score ≥ 3) [18]. For patients with ileo–ileal anastomoses, among whom no standardized scoring system exists, endoscopic recurrence was assessed via enteroscopy, with findings evaluated by adapting the principles of the modified Rutgeerts score as a reference framework [19, 20]. When existing scoring was unavailable, the most recent endoscopy report and associated images were reviewed by experienced gastroenterologists from the study team to assess recurrence status. Clinical Recurrence was determined by the Crohn’s disease activity index (CDAI) score of at least 150, indicating an active disease period, along with imaging evidence demonstrating active CD changes such as new stenosis, obstruction, or perforation. All imaging findings underwent verification by professional radiologists or IBD specialists.

### Statistical analysis

Statistical descriptions of baseline clinical characteristics were performed, while pathological features were summarized according to scoring grade. Continuous variables underwent normality testing using the Shapiro–Wilk method. Normally distributed variables were expressed as mean ± standard deviation and compared with Student’s *t*-test. Non-normally distributed variables were reported as median (interquartile range, IQR) and compared with the Kruskal–Wallis test. Categorical variables were presented as frequency (percentage) and analyzed using chi-square or Fisher’s exact tests.

Given the absence of established histological cut-off values for fibrosis in CD surgical specimens, the cohort was divided into three groups (low, moderate, high) based on data-driven tertiles of the fibrosis proportion distribution. This stratification was then analyzed separately for each tissue layer, with layer-specific tertile cut-off values (Table S1).

Nested multivariable Cox regression models assessed the association between transmural fibrosis degree and postoperative endoscopic/clinical recurrence risk, adjusting for confounders per ECCO guidelines. Three progressively adjusted models were constructed: Model 1 adjusted for age and sex; Model 2 adjusted for age, sex, smoking, behavior, past surgical history; Model 3 adjusted for age, sex, smoking, behavior, previous intestinal surgery history, granuloma at both the lesion sites and resection margins, plexitis noted in these regions, and active inflammation at the resection margins. The results were expressed as hazard ratios (HR) with 95% confidence intervals (CI).

Trend tests evaluated recurrence risk changes across increasing fibrosis grades. Kaplan–Meier analysis with log-rank testing compared survival curves between fibrosis groups. Restricted cubic splines (RCS) further characterized the dose–response relationship between fibrosis degree and outcomes. Furthermore, the relationship between fibrosis and recurrence at each distinct intestinal layer was evaluated. Subgroup analyses were conducted stratified by age, gender, and penetrating disease phenotype. Sensitivity analyses employed multivariable adjustment for variables identified with *p* < 0.1 in preliminary screening by univariate Cox regression.

All analysis procedures were performed using R 4.3.0, employed two-sided tests, and considered *p* < 0.05 statistically significant.

### Inclusion and ethics

This study was granted approval by the ethical review board of the Ruijin Hospital affiliated with Shanghai Jiao Tong University (No. 2024559). All patients provided written consent for the precision histopathology in multicentric CD disease: a multicenter bidirectional cohort study trial (ChiCTR2500099305).

## Results

### Study population

The final study cohort comprised 268 CD patients with prior intestinal surgery (Fig. 1). Participants had a mean age of 36.94 years (range from 29.11 to 46.85 years) and median disease duration of 70.20 months (range from 25.25 to 121.89 months) at the time of operation (Table 1). Demographic analysis revealed 64.2% were male and 35.8% were female. Only patients with CD involving the ileum were enrolled. 57.5% of the patients had lesions localized to the terminal ileum, while 42.5% had the ileocolonic phenotype (Table 1). Most cases were without digestive tract involvement (94.0%), perianal lesions (80.2%), or abdominal abscess complications (91.4%) (Table 1). 90.3% of the patients were non-smokers, and 63.4% did not have prior intestinal surgery (Table 1). Laboratory parameters showed normal albumin levels in 66.0% of patients and normal C-reactive protein levels in 61.2%. Preoperative medication exposure included 5-aminosalicylic acid (53.0%), steroid (26.1%), immunosuppressants (38.4%), and biologics (26.1%) (Table 1).

**Table 1:** Baseline characteristics

Characteristics	Overall	Endoscopic recurrence			Clinical recurrence		
	(*N* = 268)	No (*N* = 184)	Yes (*N* = 84)	*p*-Value	No (*N* = 177)	Yes (*N* = 91)	*p*-Value
Age (year, median (IQR))	36.94 (29.11, 46.85)	38.13 (30.30, 47.97)	35.88 (27.92, 45.81)	0.129	35.93 (29.73, 45.18)	40.34 (28.85, 48.52)	0.364
Male (%)	172 (64.2)	122 (66.3)	50 (59.5)	0.349	124 (70.1)	48 (52.7)	0.008
BMI (kg/m^2^, median (IQR))	18.61 (16.87, 20.69)	18.59 (16.86, 20.83)	18.64 (16.91, 20.52)	0.666	18.83 (17.04, 20.81)	18.07 (16.56, 20.32)	0.091
Disease duration (month, median (IQR))	70.20 (25.25, 121.89)	76.80 (27.78, 132.60)	63.23 (23.20, 99.19)	0.046	68.13 (23.73, 127.13)	75.33 (33.25, 114.22)	0.605
Age at diagnosis (%)				0.513			0.076
*A*1	8 (3.0)	4 (2.2)	4 (4.8)		6 (3.4)	2 (2.2)	
*A*2	175 (65.3)	121 (65.8)	54 (64.3)		123 (69.5)	52 (57.1)	
*A*3	85 (31.7)	59 (32.1)	26 (31.0)		48 (27.1)	37 (40.7)	
Location (%)				0.020			1.000
*L*1	154 (57.5)	115 (62.5)	39 (46.4)		102 (57.6)	52 (57.1)	
*L*3	114 (42.5)	69 (37.5)	45 (53.6)		75 (42.4)	39 (42.9)	
Upper GI tract involvement (%)	16 (6.0)	11 (6.0)	5 (6.0)	1.000	11 (6.2)	5 (5.5)	1.000
Behavior (%)				0.529			0.512
Non-B3	186 (69.4)	125 (67.9)	61 (72.6)		120 (67.8)	66 (72.5)	
B3	82 (30.6)	59 (32.1)	23 (27.4)		57 (32.2)	25 (27.5)	
Perianal involvement (%)	53 (19.8)	36 (19.6)	17 (20.2)	1.000	34 (19.2)	19 (20.9)	0.870
Smoking (%)				0.049			0.218
No	242 (90.3)	162 (88.0)	80 (95.2)		157 (88.7)	85 (93.4)	
Yes	22 (8.2)	20 (10.9)	2 (2.4)		18 (10.2)	4 (4.4)	
NA	4 (1.5)	2 (1.1)	2 (2.4)		2 (1.1)	2 (2.2)	
Intra-abdominal abscess (%)	23 (8.6)	15 (8.2)	8 (9.5)	0.891	18 (10.2)	5 (5.5)	0.287
Past surgical history (%)	98 (36.6)	68 (37.0)	30 (35.7)	0.953	59 (33.3)	39 (42.9)	0.162
Surgical procedure (%)				0.105			0.199
Non-ileocecum	162 (60.4)	119 (64.7)	43 (51.2)		101 (57.1)	61 (67.0)	
Ileocecum	104 (38.8)	64 (34.8)	40 (47.6)		74 (41.8)	30 (33.0)	
NA	2 (0.7)	1 (0.5)	1 (1.2)		2 (1.1)	0 (0.0)	
Enterostomy (%)				0.846			0.889
No	230 (85.8)	158 (85.9)	72 (85.7)		152 (85.9)	78 (85.7)	
Yes	36 (13.4)	25 (13.6)	11 (13.1)		24 (13.6)	12 (13.2)	
NA	2 (0.7)	1 (0.5)	1 (1.2)		1 (0.6)	1 (1.1)	
Anastomosis technique (%)				0.845			0.852
Non-side-to-side	62 (23.1)	43 (23.4)	19 (22.6)		42 (23.7)	20 (22.0)	
Side-to-side	204 (76.1)	140 (76.1)	64 (76.2)		134 (75.7)	70 (76.9)	
NA	2 (0.7)	1 (0.5)	1 (1.2)		1 (0.6)	1 (1.1)	
Albumin level (%)				0.701			0.331
Normal	177 (66.0)	125 (67.9)	52 (61.9)		124 (70.1)	53 (58.2)	
Mild decrease	49 (18.3)	33 (17.9)	16 (19.0)		29 (16.4)	20 (22.0)	
Moderate decrease	34 (12.7)	20 (10.9)	14 (16.7)		20 (11.3)	14 (15.4)	
Severe decrease	5 (1.9)	4 (2.2)	1 (1.2)		3 (1.7)	2 (2.2)	
NA	3 (1.1)	2 (1.1)	1 (1.2)		1 (0.6)	2 (2.2)	
CRP level (%)				0.962			0.012
Normal	164 (61.2)	113 (61.4)	51 (60.7)		119 (67.2)	45 (49.5)	
Elevated	68 (25.4)	47 (25.5)	21 (25.0)		40 (22.6)	28 (30.8)	
NA	36 (13.4)	24 (13.0)	12 (14.3)		18 (10.2)	18 (19.8)	
Pre-surgery medication (%)							
5-ASA	142 (53.0)	95 (51.6)	47 (56.0)	0.599	94 (53.1)	48 (52.7)	1.000
Steroid	70 (26.1)	45 (24.5)	25 (29.8)	0.443	42 (23.7)	28 (30.8)	0.273
Immune modulator	103 (38.4)	71 (38.6)	32 (38.1)	1.000	63 (35.6)	40 (44.0)	0.230
Biologics	70 (26.1)	48 (26.1)	22 (26.2)	1.000	46 (26.0)	24 (26.4)	1.000
Post-surgery medication (%)				0.118			0.981
No	37 (13.8)	30 (16.3)	7 (8.3)		25 (14.1)	12 (13.2)	
Yes	231 (86.2)	154 (83.7)	77 (91.7)		152 (85.9)	79 (86.8)	
Mucosal fibrosis level at lesion (%)				< 0.001			< 0.001
Low	89 (33.2)	71 (38.6)	18 (21.4)		75 (42.4)	14 (15.4)	
Moderate	88 (32.8)	65 (35.3)	23 (27.4)		63 (35.6)	25 (27.5)	
High	91 (34.0)	48 (26.1)	43 (51.2)		39 (22.0)	52 (57.1)	
Submucosal fibrosis level at lesion (%)				< 0.001			< 0.001
Low	89 (33.2)	71 (38.6)	18 (21.4)		76 (42.9)	13 (14.3)	
Moderate	89 (33.2)	68 (37.0)	21 (25.0)		59 (33.3)	30 (33.0)	
High	90 (33.6)	45 (24.5)	45 (53.6)		42 (23.7)	48 (52.7)	
Muscularis propria fibrosis level at lesion (%)				0.001			< 0.001
Low	88 (32.8)	73 (39.7)	15 (17.9)		75 (42.4)	13 (14.3)	
Moderate	89 (33.2)	58 (31.5)	31 (36.9)		62 (35.0)	27 (29.7)	
High	91 (34.0)	53 (28.8)	38 (45.2)		40 (22.6)	51 (56.0)	
Transmural fibrosis level at lesion (%)				< 0.001			< 0.001
Low	89 (33.2)	75 (40.8)	14 (16.7)		79 (44.6)	10 (11.0)	
Moderate	89 (33.2)	59 (32.1)	30 (35.7)		57 (32.2)	32 (35.2)	
High	90 (33.6)	50 (27.2)	40 (47.6)		41 (23.2)	49 (53.8)	
Mucosal fibrosis level at resection margin (%)				< 0.001			0.022
Low	91 (34.0)	72 (39.1)	19 (22.6)		68 (38.4)	23 (25.3)	
Moderate	87 (32.5)	64 (34.8)	23 (27.4)		59 (33.3)	28 (30.8)	
High	90 (33.6)	48 (26.1)	42 (50.0)		50 (28.2)	40 (44.0)	
Submucosal fibrosis level at resection margin (%)				< 0.001			0.001
Low	88 (32.8)	70 (38.0)	18 (21.4)		61 (34.5)	27 (29.7)	
Moderate	89 (33.2)	66 (35.9)	23 (27.4)		69 (39.0)	20 (22.0)	
High	91 (34.0)	48 (26.1)	43 (51.2)		47 (26.6)	44 (48.4)	
Muscularis propria fibrosis level at resection margin (%)				0.126			0.013
Low	87 (32.5)	60 (32.6)	27 (32.1)		59 (33.3)	28 (30.8)	
Moderate	90 (33.6)	68 (37.0)	22 (26.2)		68 (38.4)	22 (24.2)	
High	91 (34.0)	56 (30.4)	35 (41.7)		50 (28.2)	41 (45.1)	
Transmural fibrosis level at resection margin (%)				< 0.001			< 0.001
Low	87 (32.5)	68 (37.0)	19 (22.6)		68 (38.4)	19 (20.9)	
Moderate	90 (33.6)	68 (37.0)	22 (26.2)		65 (36.7)	25 (27.5)	
High	91 (34.0)	48 (26.1)	43 (51.2)		44 (24.9)	47 (51.6)	
Granuloma at lesion (%)	148 (55.2)	82 (44.6)	66 (78.6)	< 0.001	67 (37.9)	81 (89.0)	< 0.001
Granuloma at resection margin (%)	75 (28.0)	42 (22.8)	33 (39.3)	0.008	26 (14.7)	49 (53.8)	< 0.001
Plexitis at lesion (%)	213 (79.5)	136 (73.9)	77 (91.7)	0.001	124 (70.1)	89 (97.8)	< 0.001
Plexitis at resection margin (%)	121 (45.1)	73 (39.7)	48 (57.1)	0.011	58 (32.8)	63 (69.2)	< 0.001
Active inflammation at resection margin (%)	56 (20.9)	35 (19.0)	21 (25.0)	0.340	33 (18.6)	23 (25.3)	0.269
Adler fibrosis degree at lesion site (%)				< 0.001			< 0.001
Level 1	19 (7.1)	18 (9.8)	1 (1.2)		19 (10.7)	0 (0.0)	
Level 2	70 (26.1)	60 (32.6)	10 (11.9)		65 (36.7)	5 (5.5)	
Level 3	125 (46.6)	77 (41.8)	48 (57.1)		78 (44.1)	47 (51.6)	
Level 4	54 (20.1)	29 (15.8)	25 (29.8)		15 (8.5)	39 (42.9)	
Adler fibrosis degree at resection margin (%)				< 0.001			< 0.001
Level 1	104 (38.8)	81 (44.0)	23 (27.4)		81 (45.8)	23 (25.3)	
Level 2	120 (44.8)	84 (45.7)	36 (42.9)		82 (46.3)	38 (41.8)	
Level 3	41 (15.3)	17 (9.2)	24 (28.6)		13 (7.3)	28 (30.8)	
Level 4	3 (1.1)	2 (1.1)	1 (1.2)		1 (0.6)	2 (2.2)	

### Fibrosis degree independently associated with recurrence risk

**Fig. 2:**
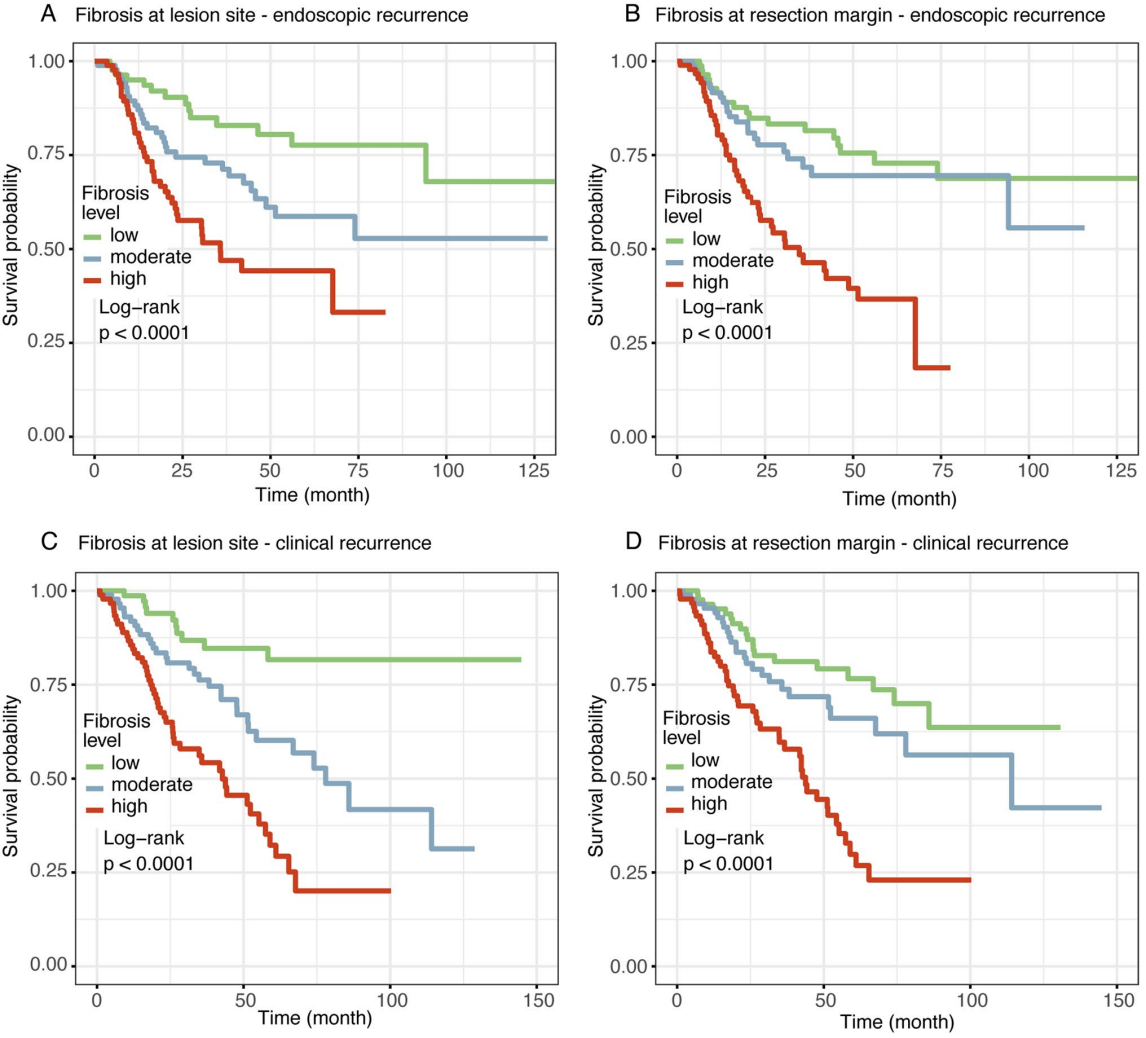
The relationship between the fibrosis degree and postoperative recurrence risk**. A** Association between the degree of transmural fibrosis at the lesion site and postoperative endoscopic recurrence in CD. **B** Association between the degree of transmural fibrosis at the resection margin site and postoperative endoscopic recurrence in CD. **C** Association between the degree of transmural fibrosis at the lesion site and postoperative clinical recurrence in CD. **D** Association between the degree of transmural fibrosis at the resection margin site and postoperative clinical recurrence in CD. In all panels, the green curve represents low-level fibrosis, the blue curve represents moderate-level fibrosis, and the red curve represents high-level fibrosis

A total of 84 patients were observed to have endoscopic recurrence while 91 patients were observed to have clinical recurrence. The actual distribution of fibrosis ratios is continuous (Fig. S3). Patients were categorized into low, moderate, and high fibrosis groups based on tertiles of this distribution (Table S1, Fig. S4). Survival analysis demonstrated significant positive associations between transmural intestinal fibrosis severity at both lesion site and resection margin with increased risks of endoscopic recurrence and clinical recurrence. Higher fibrosis degrees correlated with progressively elevated endoscopic and clinical recurrence hazards (Fig. 2). Multivariable Cox regression confirmed that each standard deviation (SD) increase in lesion site fibrosis raised endoscopic recurrence risk with a HR of 1.46 (95% CI 1.18–1.80, *p* = 0.001), while moderate and high fibrosis exhibited HRs of 2.27 (95% CI 1.17–4.39, *p* = 0.015) and 3.02 (95% CI 1.61–5.66, *p* = 0.001) respectively versus low fibrosis, showing significant trend effects (*p* = 0.001) (Table 2). Margin site fibrosis similarly increased endoscopic recurrence risk per SD (HR = 1.35, 95% CI 1.12–1.63; *p* = 0.002). Compared with low fibrosis at the resection margin, the HR for endoscopic recurrence was 1.30 (95% CI 0.69–2.44, *p* = 0.422) for moderate fibrosis and 2.88 (95% CI 1.63–5.09, *p* < 0.001) for high fibrosis (*p* < 0.001) (Table 2). Clinical recurrence analysis revealed parallel associations with lesion site fibrosis (HR = 1.95, 95% CI 1.59–2.39; *p* < 0.001) and resection margin fibrosis (HR = 1.29, 95% CI 1.09–1.54; *p* = 0.003). The progressive risk increased with higher fibrosis grades remained significant (*p* < 0.001) (Table 2).

**Table 2:** The nested multifactorial Cox regression analysis for transmural fibrosis and postoperative recurrence

Outcome	Transmural fibrosis	Model 0		Model 1		Model 2		Model 3	
		HR (95% CI)	*p*-Value	HR (95% CI)	*p*-Value	HR (95% CI)	*p*-Value	HR (95% CI)	*p*-Value
Endoscopic recurrence	Lesion site (per SD increase)	1.65 (1.34, 2.03)	< 0.001	1.65 (1.34, 2.04)	< 0.001	1.62 (1.30, 2.00)	< 0.001	1.46 (1.18, 1.80)	0.001
	Lesion site (moderate vs. low)	2.13 (1.13, 4.03)	0.020	2.12 (1.12, 4.00)	0.021	2.08 (1.09, 3.97)	0.027	2.27 (1.17, 4.39)	0.015
	Lesion site (high vs. low)	3.80 (2.05, 7.07)	< 0.001	3.63 (1.95, 6.75)	< 0.001	3.43 (1.83, 6.43)	< 0.001	3.02 (1.61, 5.66)	0.001
	*p*.trend		< 0.001		< 0.001		< 0.001		0.001
	Resection margin (per SD increase)	1.50 (1.26, 1.79)	< 0.001	1.46 (1.23, 1.74)	< 0.001	1.43 (1.20, 1.70)	< 0.001	1.35 (1.12, 1.63)	0.002
	Resection margin (moderate vs. low)	1.35 (0.73, 2.50)	0.340	1.40 (0.75, 2.59)	0.287	1.28 (0.68, 2.40)	0.443	1.30 (0.69, 2.44)	0.422
	Resection margin (high vs. low)	3.33 (1.92, 5.77)	< 0.001	3.40 (1.96, 5.90)	< 0.001	3.17 (1.82, 5.52)	< 0.001	2.88 (1.63, 5.09)	< 0.001
	*p*.trend		< 0.001		< 0.001		< 0.001		< 0.001
Clinical recurrence	Lesion site (per SD increase)	2.11 (1.74, 2.56)	< 0.001	2.16 (1.77, 2.63)	< 0.001	2.19 (1.79, 2.68)	< 0.001	1.95 (1.59, 2.39)	< 0.001
	Lesion site (moderate vs. low)	3.22 (1.58, 6.55)	0.001	3.21 (1.57, 6.54)	0.001	3.51 (1.70, 7.24)	0.001	3.62 (1.75, 7.52)	0.001
	Lesion site (high vs. low)	6.80 (3.40, 13.59)	< 0.001	6.93 (3.46, 13.90)	< 0.001	7.30 (3.61, 14.78)	< 0.001	5.91 (2.89, 12.06)	< 0.001
	*p*.trend		< 0.001		< 0.001		< 0.001		< 0.001
	Resection margin (per SD increase)	1.48 (1.26, 1.73)	< 0.001	1.48 (1.26, 1.74)	< 0.001	1.45 (1.24, 1.71)	< 0.001	1.29 (1.09, 1.54)	0.003
	Resection margin (moderate vs. low)	1.59 (0.88, 2.90)	0.126	1.56 (0.86, 2.83)	0.146	1.46 (0.80, 2.69)	0.221	1.30 (0.70, 2.41)	0.400
	Resection margin (high vs. low)	3.71 (2.16, 6.37)	< 0.001	3.68 (2.14, 6.34)	< 0.001	3.62 (2.09, 6.29)	< 0.001	2.85 (1.62, 5.01)	< 0.001
	*p*.trend		< 0.001		< 0.001		< 0.001		< 0.001

Model 0, crude; Model 1, adjusted with age, sex; Model 2, adjusted with age, sex, smoking, behavior, past surgical history; Model 3, adjusted with age, sex, smoking, behavior, past surgical history, total granuloma, total plexitis, active inflammation at resection margin

**Fig. 3:**
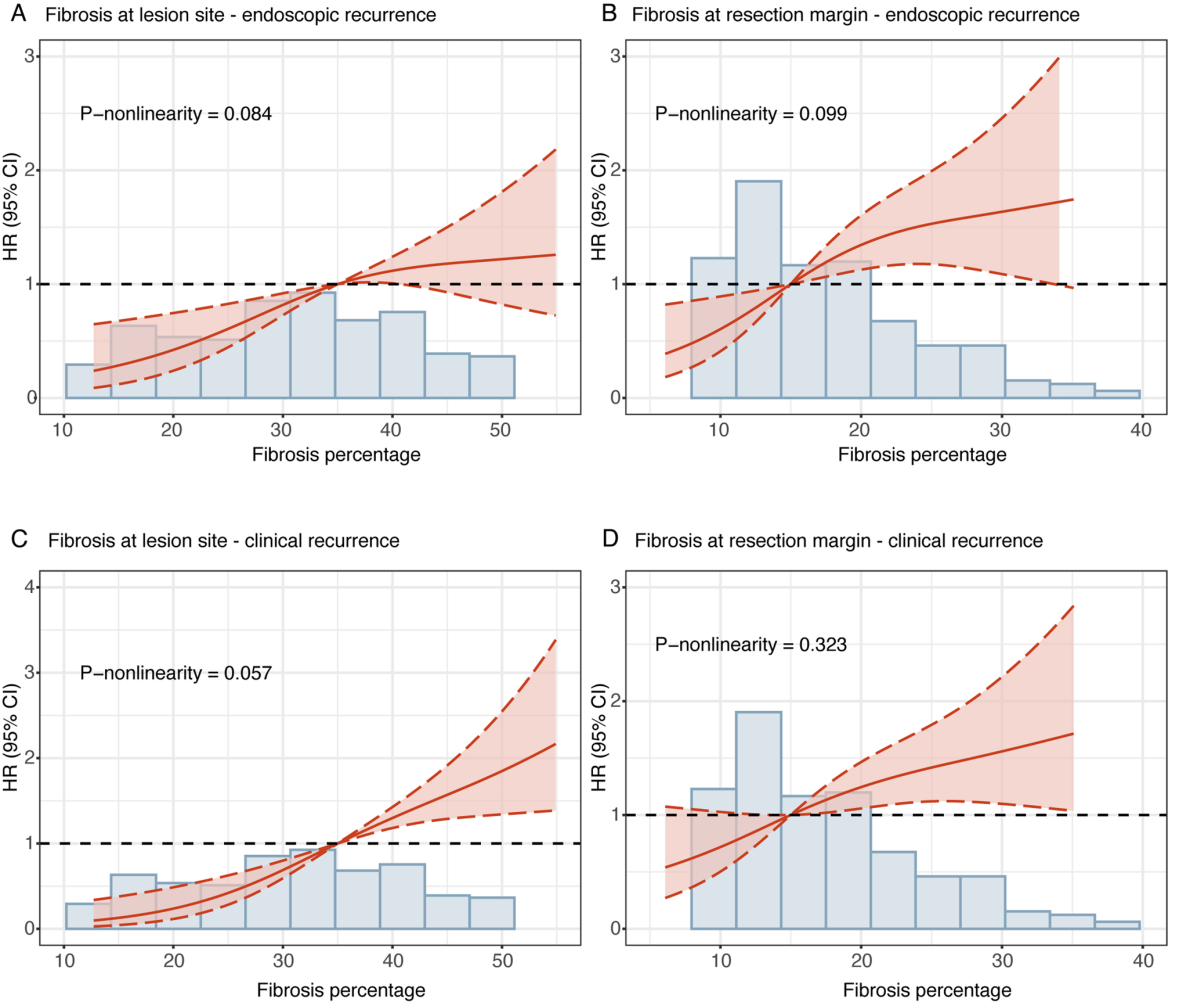
Nonlinear association between the fibrosis degree and postoperative recurrence risk. **A** Association between the degree of transmural fibrosis at the lesion site and endoscopic recurrence. **B** Association between the degree of transmural fibrosis at the resection margin site and endoscopic recurrence. Analyses for **A** and **B** were adjusted for age, disease duration, disease location, smoking status, ileocolonic resection, presence of granulomas, and presence of plexitis. **C** Association between the degree of transmural fibrosis at the lesion site and clinical recurrence. **D** Association between the degree of transmural fibrosis at the resection margin site and clinical recurrence. Analyses for **C** and **D** were adjusted for sex, body mass index (BMI), presence of intra-abdominal abscess, history of prior intestinal surgery, hematocrit level, preoperative biologic use, postoperative steroid use, postoperative methotrexate use, presence of granulomas, and presence of plexitis

**Fig. 4:**
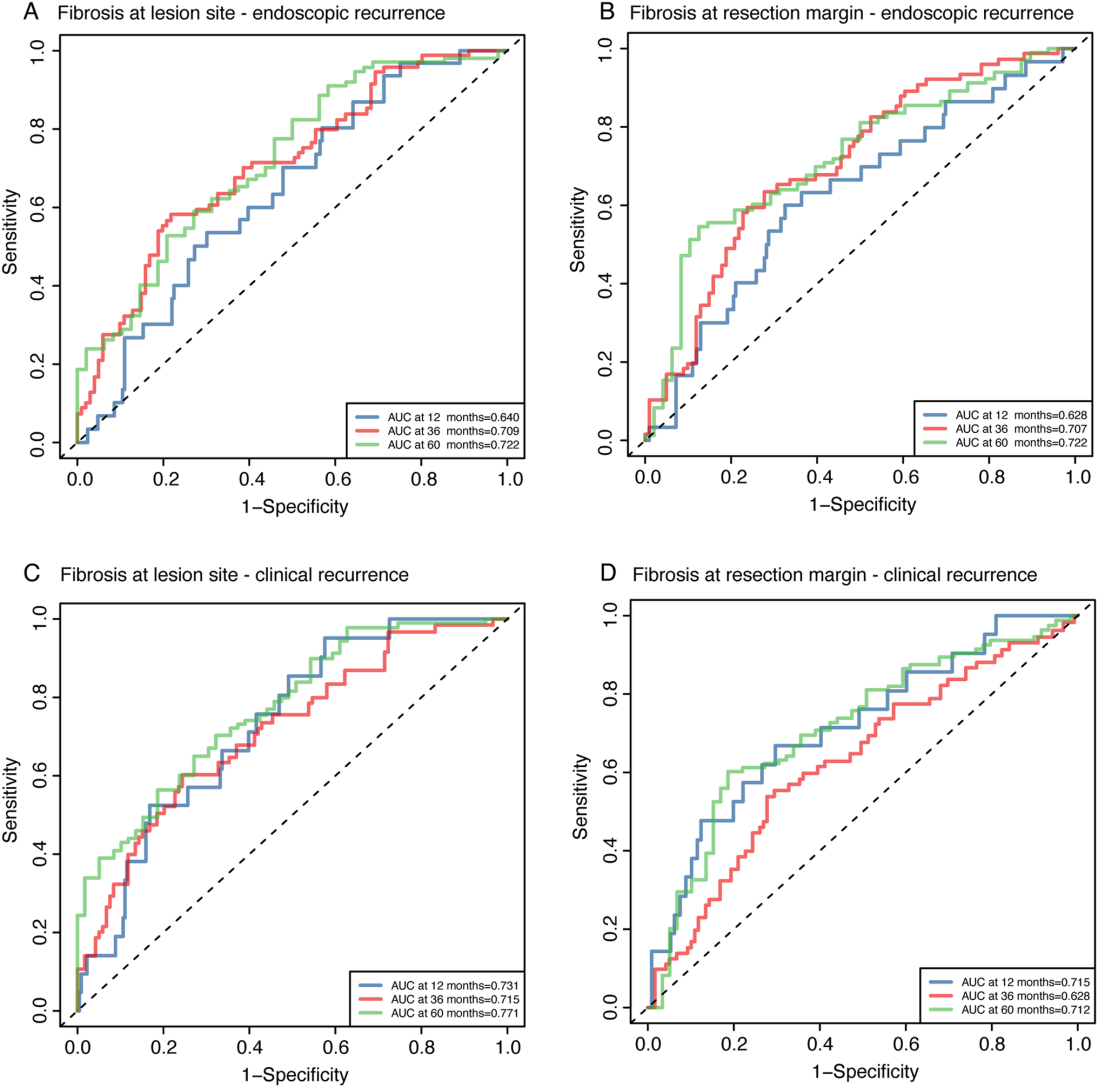
The AUC of fibrosis to predict postoperative recurrence risk. **A** Predictive value of transmural fibrosis at the lesion site for endoscopic recurrence. **B** Predictive value of transmural fibrosis at the resection margin site for endoscopic recurrence. **C** Predictive value of transmural fibrosis at the lesion site for clinical recurrence. **D** Predictive value of transmural fibrosis at the resection margin site for clinical recurrence. In all panels the AUC values representing predictive performance are displayed for 1 year (Blue), 3 years (Red), and 5 years (Green)

RCS analysis established monotonic dose–response relationships between fibrosis severity and recurrence risks without evidence of nonlinearity (Fig. 3). Consistent associations were observed when analyzing mucosal, submucosal, and muscularis propria fibrosis separately (Table S2). Fibrosis at both the lesion site and the resection margin site demonstrated significant predictive value for postoperative endoscopic (area under the curve, AUC: 0.722 for both sites) and clinical recurrence (AUCs: 0.771 and 0.712 at the lesion and margin sites, respectively) (Fig. 4). In aggregate, the results supported that an increased degree of fibrosis at the lesion site and the resection margin independently associated with an elevated risk of endoscopic recurrence and clinical recurrence after CD surgery.

### Robustness of fibrosis-recurrence association

Subgroup analyses in patients stratified by age, gender, and penetrating disease phenotype maintained consistent fibrosis-recurrence associations (Tables S3–S6). In addition, we independently evaluated histopathological fibrosis at the proximal and distal margins through subgroup analysis. The results demonstrated that transmural fibrosis at both resection margins was significantly associated with an increased risk of postoperative recurrence (Table S7). Subsequently, we screened the variables with a significant *p* < 0.1 through univariate Cox regression analysis (Table S8) and constructed a multivariate adjusted Cox regression analysis to further explore the association between the degree of transmural fibrosis and postoperative recurrence of CD. Multivariable models adjusted for covariates including age, lesion location, smoking history, ileocolic resection, prior surgery, granuloma, and plexitis confirmed the independent predictive value of transmural fibrosis for postoperative endoscopic recurrence (Tables S8, S9). Lesion site fibrosis per SD increase maintained an adjusted HR of 1.63 (95% CI 1.29–2.05; *p* < 0.001) while resection margin fibrosis showed HR of 1.50 (95% CI 1.25–1.80; *p* < 0.001). Notably, increasing fibrosis severity correlates with progressively higher recurrence risks. Clinical recurrence analysis yielded similar robust associations after adjustment for gender, smoking, preoperative use of immune agents, preoperative use of biological agents, granuloma, and plexitis, with lesion site fibrosis HR of 2.21 (95% CI 1.78–2.75; *p* < 0.001) and resection margin fibrosis HR of 1.51 (95% CI 1.28–1.77; *p* < 0.001) (Tables S8, S9). The graded increase in recurrence risk across fibrosis severity tertiles remained statistically significant in all models (*p* < 0.001), confirming the stable relationship between elevated fibrosis burden and postoperative recurrence risks.

## Discussion

Postoperative recurrence remains a frequent and clinically significant challenge for CD patients following intestinal resection. Acknowledging the limitations of existing postoperative management and prevention approaches, our research established a multicenter cohort to investigate the relationship between intestinal fibrosis severity and postoperative recurrence in CD. We were the first to propose that transmural fibrosis at both the primary lesion site and the resection margin independently predicted increased risk of postoperative endoscopic recurrence and clinical recurrence, suggesting that fibrosis assessment may offer superior risk stratification.

This study established a multi-center retrospective cohort of postoperative recurrence in CD. Unlike previous limited single-center analyses that focused solely on radiographic outcomes in anti-TNFα-treated patients [21], we systematically examined both endoscopic and clinical recurrence across diverse patient populations. The multi-center design substantially mitigated the inherent biases and enhanced the generalizability of our findings. By establishing standardized fibrosis quantification across three major referral centers created a robust clinical research framework that addresses prior limitations in sample size and methodological consistency. The multi-center cohort of postoperative recurrence in CD that we constructed laid a solid data foundation for subsequent studies.

This study provides the first definitive evidence that transmural fibrosis severity serves as an independent predictor for multiple recurrence endpoints, offering clinicians a valuable new tool for postoperative risk stratification. In recent years, histological analysis of resected specimens has been utilized to predict postoperative recurrence of CD. Nevertheless, intestinal fibrosis, one of the main causes of CD disease alterations and surgical intervention, has not been thoroughly investigated. A recent multi-center prospective study demonstrated that combining histopathological features (including inflammation at resection margins) with clinical risk factors improved prediction accuracy for severe endoscopic recurrence (AUC = 0.79) [22], suggesting the potential predictive value of histopathological features. However, this study did not focus on the predictive role of fibrosis. Whether fibrosis is a risk factor for postoperative recurrence of CD remains controversial. A report published in 1990 did not identify any association between histological features of the resection margin, including fibrosis, and postoperative endoscopic recurrence [23]. However, Hammoudi et al*.* discovered in a prospective multi-center study of 211 patients undergoing ileocolic resection that submucosal fibrosis at the resection margin was the most common submucosal lesion (33%) and was significantly associated with endoscopic recurrence (OR = 1.80; 95% CI 1.01, 3.21; *p* = 0.04) [24]. Our study examined established and emerging risk factors within a dedicated recurrence cohort, revealing that transmural fibrosis at both lesion site and resection margin constitutes an independent risk factor for endoscopic recurrence and clinical recurrence, with recurrence risk escalating alongside fibrosis severity. These associations remained robust in sensitivity analyses. Fibrosis severity was quantified as a continuous proportion-based variable and categorized using a tertile-based approach. This strategy was adopted due to the absence of established or clinically validated histological cut-off values for fibrosis severity in CD surgical specimens. Figure S5 illustrates the stratification of intestinal fibrosis, showing the distribution of fibrotic burden across the transmural wall as well as within the individual mucosal, submucosal, and muscularis propria layers. While this data-driven approach allowed for internal comparisons, the resulting cutoff values are cohort-specific and require validation in independent populations before potential clinical application. The RCS analysis visually confirmed a generally monotonic, dose–response relationship between the severity of fibrosis and the risk of postoperative recurrence (for both endoscopic and clinical outcomes), with a notably steeper increase in hazard ratios within the ‘High’ fibrosis range. Quantification of fibrosis employed the collagen area fraction index method described and validated by Li et al*.* [16], which correlates well with traditional histological scoring systems.

A pivotal finding of our study is that the elevated risk of postoperative recurrence was predominantly concentrated in patients within the highest tertile of histological fibrosis (‘High’ group). This pattern likely reflects biologically meaningful stage-dependent differences in fibrotic remodeling. Moderate fibrosis may represent a transitional or heterogeneous state, in which extracellular matrix deposition and stromal activation have not yet reached a threshold associated with stable architectural distortion and irreversible tissue remodeling. In contrast, high fibrosis is more likely to reflect advanced, consolidated fibrotic remodeling accompanied by persistent stromal–immune crosstalk, thereby conferring a disproportionately higher and clinically actionable recurrence risk. The concentration of risk within the highest fibrosis tertile has direct clinical utility. It suggests that classifying patients into this ‘High’ group after surgery offers a practical method for postoperative risk stratification. This subgroup may represent the most relevant target for intensified prophylactic strategies. Although advanced fibrosis reflects structural remodeling, it remains partly driven by persistent inflammatory signaling. Biologic therapies targeting TNF or IL-12/23 have demonstrated benefit in stricturing phenotypes and may modulate fibrogenic pathways [25–27]. In parallel, novel antifibrotic agents, including tulisokibart, and obefazimod, highlight a growing therapeutic focus on directly reversing structural remodeling [28, 29]. Emerging evidence further suggests that advanced fibrotic remodeling may be associated with attenuated responses to standard postoperative therapies [30, 31], underscoring the importance of risk-adapted management. Therefore, defining the upper tertile as a high-risk group may facilitate risk-adapted intervention. In this population, early biologic optimization or antifibrotic therapy represents a promising therapeutic strategy.

Crucially, while previous studies have primarily focused on the histological features of resection margins, we uniquely established an independent association between fibrosis at the primary lesion site and postoperative recurrence risk. Compared to fibrosis at the margin, fibrosis at the disease site demonstrated equally predictive value for both postoperative endoscopic (AUC = 0.722) and clinical recurrence (AUC = 0.771, which was greater than 0.712 for the margin), indicating its role not merely as a marker of disease severity but as a key prognostic indicator. Compared to European and North American cohorts, our study observed lower recurrence rates [6, 7], potentially attributable to population phenotypic diversity [32], cohort-specific characteristics (exclusion of patients with prior resections), low smoking prevalence, and high rates of standardized postoperative prophylaxis. This study also examined other pathological and clinical characteristics for their association with recurrence. Univariate analysis linked postoperative endoscopic recurrence to granuloma, plexitis, age, disease duration, lesion location, and ileocolic resection, while clinical recurrence was associated with granuloma, plexitis, body mass index, abdominal abscess, preoperative biologic agent use, and postoperative steroid use. The associations of granuloma and plexitis with recurrence risk align with previous studies [9].

This study further explored the debate regarding whether pathological outcomes differ between proximal and distal resection margins. Through subgroup analysis, we confirmed that transmural fibrosis at either margin serves as an independent predictor of postoperative recurrence. While distinguishing between margins may hold significance in specific surgical or mechanistic research, the similar predictive strength observed in our cohort supports combining them into a unified variable termed “resection margin fibrosis” to facilitate broader clinical application. This consolidated variable simplifies risk stratification without compromising predictive validity, thereby enhancing its utility in guiding postoperative management decisions. Nevertheless, we acknowledge that margin-specific factors could theoretically influence outcomes, and future studies with larger cohorts may further elucidate subtle distinctions.

In defining our study cohort, we specifically focused on *L*1 and *L*3 phenotypes, excluding the *L*2 subtype due to fundamental differences in biological behavior and recurrence patterns [33]. The *L*2 subtype represented only 1.5% of our initial cohort (this data is not displayed in the present analysis), and its inclusion would contribute minimal analytical value while potentially introducing statistical bias. This decision was supported by the distinct surgical approaches required for each phenotype, specifically enterectomy or ileocecal resection for *L*1/*L*3 as opposed to colectomy for *L*2, representing different disease processes [34, 35]. More importantly, their postoperative courses differ fundamentally. While *L*1/*L*3 diseases consistently recur in the neoterminal ileum, *L*2 typically manifests as pouchitis or perianal complications [33]. Emerging evidence indicates that *L*2 constitutes a biologically distinct disease entity, characterized by unique genetic susceptibility, microbial composition, and metabolomic signatures, differentiating it from ileal-involving phenotypes [33, 36]. Thus, the exclusion of *L*2 phenotype ensures cohort homogeneity and prevents confounding of the ileal-specific recurrence under investigation.

Beyond phenotypic location, disease behavior also influences fibrotic progression. Penetrating (B3) Crohn’s disease represents a distinct disease phenotype rather than a transient inflammatory state. It reflects sustained transmural inflammation and advanced tissue injury accumulated over the disease course. In contrast, non-penetrating phenotypes (B1/B2) generally involve more superficial inflammation and may evolve into penetrating disease over time. Histopathological studies of fistulizing Crohn’s disease have shown dense and spatially organized immune cell infiltration, epithelial disruption, granulation tissue formation, and deep tissue remodeling [37–39]. These features indicate a qualitatively different and more complex inflammatory microenvironment compared with non-penetrating disease. Such sustained inflammation and structural remodeling provide a biological basis for the increased fibrosis burden and worse postoperative outcomes observed in B3 patients. Therefore, the comparison between non-B3 and B3 subgroups in this study was designed to reflect differences in cumulative inflammatory injury and depth of tissue involvement that are directly relevant to fibrosis severity.

When interpreting the findings of this study, several methodological considerations should be taken into account. First, to ensure histopathological accuracy and reliability, cases with suboptimal or incomplete histological sections had to be excluded. While this strengthened the internal validity of our pathological assessments, it introduced potential selection bias. This constraint is characteristic of retrospective studies, as researchers have no control over the original specimen acquisition and processing. The reasons for case exclusion have been transparently reported. Second, regarding endoscopic assessment, the absence of a validated scoring system for ileo-ileal anastomoses led us to pragmatically adapt the principles of the modified Rutgeerts score as a reference framework [19, 20]. While the original Rutgeerts score was designed for the ileocolonic anastomosis, our application focused on analogous endoscopic features, including aphthous ulcers, larger ulcers, and stenosis, at the anastomotic line and the pre-anastomotic small bowel segment. This approach provided a consistent, structured, and clinically grounded method to grade disease severity, ensuring comparability across all patients in our cohort. We noted that the direct extrapolation of the Rutgeerts score to a different anatomical context remains an aspect for future validation. However, this adapted use represents a necessary and reasoned strategy to address a current gap in standardized assessment tools. Third, concerning fibrosis quantification, our analysis was confined to the consistently identifiable intestinal wall layers (mucosa to muscularis propria), excluding the serosal layer due to its variable preservation across specimens. Although this restricted assessment may underestimate the total fibrotic burden in advanced transmural disease, it was essential to minimize measurement variability and ensure cohort-wide comparability. Inclusion of incomplete or variably preserved serosal tissue could introduce substantial measurement variability and compromise consistency across specimens.

Our study possessed several notable methodological and clinical advantages in understanding postoperative CD recurrence. First, as the largest multi-center investigation to specifically examine the postoperative prognostic value of histologically quantified intestinal fibrosis, it offered superior statistical power over previous studies. In addition, our standardized, quantitative digital pathology methods provided objective fibrosis assessment across all intestinal layers, overcoming the subjectivity limitations of traditional scoring methods. Third, we comprehensively evaluated both endoscopic and clinical recurrence endpoints for complete disease progression assessment. Importantly, the inclusion of both lesion sites and resection margins offered unique spatial insights into fibrosis-related recurrence risk. Finally, the rigorous adjustment for multiple established clinical and pathological confounders ensured the independent predictive value of fibrosis severity. Together, these strengths provided strong evidence supporting the clinical utility of fibrosis assessment in postoperative management decisions.

Several limitations warrant acknowledgment. These include the retrospective design, which introduced potential selection bias due to the exclusion of cases with compromised histological integrity; the application of the Rutgeerts score as a reference framework for ileo-ileal anastomoses in the absence of a validated endoscopic scoring system; and the exclusion of the serosal layer from fibrosis quantification, which may underestimate the total fibrotic burden. In addition, the study faced sample size constraints despite multi-center design, and had a relatively short follow-up duration. While we established a significant association between fibrosis and recurrence, the underlying mechanisms require elucidation. Future efforts will expand this multi-center cohort through broader collaborations, extend follow-up, standardize tissue handling and endoscopic scoring systems, incorporate comprehensive serosal evaluation, and employ advanced techniques to unravel fibrotic mechanisms. Machine learning and AI-driven histopathological analysis will further explore prognostic correlations to refine predictive models for risk stratification.

In summary, the severity of intestinal fibrosis at both lesion sites and resection margins independently correlates with heightened recurrence risk, and its assessment may serve as a valuable tool for identifying high-risk patients requiring intensified postoperative management strategies.

## Supplementary Information

Below is the link to the electronic supplementary material.

Supplementary file1 (DOCX 7431 KB)
