# Supplementary material for: Severity of surgical histopathological fibrosis predicted postoperative recurrence in Crohn’s disease: a multi-center retrospective cohort study
Source: J Gastroenterol. 2026 Feb 26;61(6):750–64. doi: 10.1007/s00535-026-02374-9 (PMC13219220; doi:10.1007/s00535-026-02374-9)
Supplement: Supplementary file 1 — Supplementary file1 (DOCX 7431 KB) [file 535_2026_2374_MOESM1_ESM.docx]

**Severity of Surgical Histopathological Fibrosis Predicted Postoperative Recurrence in Crohn's Disease: A Multi-center Retrospective Cohort Study**

**Journal of Gastroenterology**

Xinyu Wang^1, †^, Yiwen Tu^1, †^, Shuowen Zhang^1, †^, Tianyi Che^1^, Shenglan You^1^, Weitong Gao^1^, Lingying Zhao^1^, Ren Mao^2^, Jing Sun^1^, Yubei Gu^1^, Yao Zhang^1^, Zirui He^3, *^, Yi Li^4, *^, Duowu Zou^1, *^

**^*^ Corresponding author:**
Duowu Zou, Department of Gastroenterology, Ruijin Hospital Affiliated to Shanghai Jiao Tong University School of Medicine, 197 Ruijin 2nd Road, Shanghai 200025, China. Telephone: +86-21-64370045; Email: zdwrjxh66@sjtu.edu.cn

Yi Li, Department of General Surgery, Jinling Hospital, Nanjing University School of Medicine, No. 305 East Zhongshan Road, Nanjing 210002, Jiangsu Province, China. Telephone: +86-25-80860114; Email: liyi.jlh@hotmail.com

Zirui He, Department of General Surgery, Ruijin Hospital, School of Medicine, 197 Ruijin 2nd Road, Shanghai 200025, China. Telephone: +86-21-64370045; Email: hezirui@aliyun.com

**^†^Xinyu Wang, Yiwen Tu, and Shuowen Zhang contributed equally to this work**

**Supplementary materials**

**Materials and methods**

**Ascertainment of intestinal fibrosis**

The degree of intestinal fibrosis was evaluated by software recognition of the proportion of collagen fiber area in Masson-stained tissue sections. The scanned Masson-stained electronic sections were opened in the Olympus Image Viewer electronic image reading software, and the magnification was adjusted to display the entire intestinal specimen. The image was exported in Tiff format. Additionally, the window was adjusted to the mucosal area and muscularis propria area, and images were exported at a magnification of 10 times; the window was adjusted to the submucosal area, and the image was exported at a magnification of 4 times. The exported Tiff format images were opened in the Imagine Pro Plus software. Researchers utilized tools to delineate the tissue contours and selected blue pixels (collagen fibers stained blue) in the image with a color picker. The software would automatically mark all pixels with similar colors. The area calculation function of the software was employed to automatically calculate the area of the delineated intestinal tissue specimen, the total area of the blue-marked collagen fibers, and the proportion of the total area of the collagen fibers to the area of the intestinal tissue specimen. The color extraction scheme of the software was stored, and mouse macro-operations were recorded in the software to facilitate the automatic completion of other image processing. Due to the varying degrees of staining in the sections, for individual sections with lighter or darker staining, researchers needed to manually fine-tune the color extraction scheme. The degree of intestinal fibrosis was measured by the collagen area fraction (total area of collagen fibers / area of intestinal tissue specimen × 100%)^[1]^.

**Supplementary Tables**

**Table S1. Cut-off values for transmural and layer-specific fibrosis defining low, moderate, and high groups**

| Layer | Site | Low Fibrosis^a^ | Moderate Fibrosis^b^ | High Fibrosis^c^ |
| --- | --- | --- | --- | --- |
| Mucosal | Lesion site | ≤ 0.1188 | 0.1188 – 0.1811 | > 0.1811 |
|  | Resection margin | ≤ 0.0713 | 0.0713 – 0.1167 | > 0.1167 |
| Submucosal | Lesion site | ≤ 0.2838 | 0.2838 – 0.3990 | > 0.3990 |
|  | Resection margin | ≤ 0.1517 | 0.1517 – 0.2500 | > 0.2500 |
| Muscularis propria | Lesion site | ≤ 0.0905 | 0.0905 – 0.1788 | > 0.1788 |
|  | Resection margin | ≤ 0.0532 | 0.0532 – 0.1152 | > 0.1152 |
| Transmural | Lesion site | ≤ 0.2685 | 0.2685 – 0.3699 | > 0.3699 |
|  | Resection margin | ≤ 0.1281 | 0.1281 – 0.1919 | > 0.1919 |

^a^The cut-off value for the Low Fibrosis Group is the first tertile of the cohort distribution. Cases with a fibrosis proportion less than or equal to this value are categorized as Low Fibrosis.

^b^The cut-off values for the Moderate Fibrosis Group are the first and second tertiles. Cases with a fibrosis proportion greater than the lower cut-off and less than or equal to the upper cut-off are categorized as Moderate Fibrosis.

^c^The cut-off value for the High Fibrosis Group is the second tertile. Cases with a fibrosis proportion greater than this value are categorized as High Fibrosis.

All values represent the proportion of fibrotic area relative to total tissue area. These data-driven cut-offs are specific to this cohort.

**Table S2. The nested multifactorial Cox regression analysis for sectional fibrosis and postoperative recurrence**

| **Outcome** | **Site** | **Sectional fibrosis** | **Model 0** | |  | **Model 1** | |  | **Model 2** | |  | **Model 3** | |
| --- | --- | --- | --- | --- | --- | --- | --- | --- | --- | --- | --- | --- | --- |
|  |  |  | **HR (95%CI)** | ***P*-value** |  | **HR (95%CI)** | ***P*-value** |  | **HR (95%CI)** | ***P*-value** |  | **HR (95%CI)** | ***P*-value** |
| **Endoscopic recurrence** | Lesion site | Quantified mucosal fibrosis (per SD increase) | 1.60 (1.30, 1.97) | <0.001 |  | 1.57 (1.28, 1.93) | <0.001 |  | 1.56 (1.27, 1.91) | <0.001 |  | 1.34 (1.08, 1.66) | 0.009 |
|  |  | Low mucosal fibrosis | 1.00 |  |  | 1.00 |  |  | 1.00 |  |  | 1.00 |  |
|  |  | Moderate mucosal fibrosis | 1.08 (0.58, 2.00) | 0.815 |  | 1.08 (0.58, 2.00) | 0.806 |  | 1.01 (0.54, 1.89) | 0.963 |  | 0.92 (0.49, 1.72) | 0.788 |
|  |  | High mucosal fibrosis | 2.47 (1.42, 4.29) | 0.001 |  | 2.60 (1.49, 4.54) | 0.001 |  | 2.54 (1.45, 4.45) | 0.001 |  | 1.85 (1.04, 3.31) | 0.037 |
|  |  | *p*.trend |  | <0.001 |  |  | <0.001 |  |  | <0.001 |  |  | 0.021 |
|  |  | Quantified submucosal fibrosis (per SD increase) | 1.85 (1.51, 2.28) | <0.001 |  | 1.82 (1.48, 2.24) | <0.001 |  | 1.75 (1.41, 2.18) | <0.001 |  | 1.70 (1.37, 2.11) | <0.001 |
|  |  | Low submucosal fibrosis | 1.00 |  |  | 1.00 |  |  | 1.00 |  |  | 1.00 |  |
|  |  | Moderate submucosal fibrosis | 1.34 (0.71, 2.53) | 0.363 |  | 1.34 (0.71, 2.53) | 0.367 |  | 1.26 (0.66, 2.41) | 0.476 |  | 1.22 (0.63, 2.37) | 0.548 |
|  |  | High submucosal fibrosis | 3.83 (2.18, 6.73) | <0.001 |  | 3.66 (2.08, 6.47) | <0.001 |  | 3.30 (1.83, 5.94) | <0.001 |  | 3.08 (1.68, 5.66) | <0.001 |
|  |  | *p*.trend |  | <0.001 |  |  | <0.001 |  |  | <0.001 |  |  | <0.001 |
|  |  | Quantified muscularis propria fibrosis (per SD increase) | 1.51 (1.25, 1.83) | <0.001 |  | 1.46 (1.20, 1.77) | <0.001 |  | 1.41 (1.16, 1.72) | 0.001 |  | 1.22 (1.00, 1.50) | 0.052 |
|  |  | Low muscularis propria fibrosis | 1.00 |  |  | 1.00 |  |  | 1.00 |  |  | 1.00 |  |
|  |  | Moderate muscularis propria fibrosis | 2.34 (1.26, 4.37) | 0.007 |  | 2.47 (1.32, 4.61) | 0.005 |  | 2.37 (1.26, 4.45) | 0.007 |  | 2.19 (1.17, 4.13) | 0.015 |
|  |  | High muscularis propria fibrosis | 3.50 (1.91, 6.42) | <0.001 |  | 3.36 (1.83, 6.17) | <0.001 |  | 3.01 (1.63, 5.56) | <0.001 |  | 2.43 (1.30, 4.52) | 0.005 |
|  |  | *p*.trend |  | <0.001 |  |  | <0.001 |  |  | 0.001 |  |  | 0.007 |
|  | Resection margin | Quantified mucosal fibrosis (per SD increase) | 1.47 (1.23, 1.76) | <0.001 |  | 1.48 (1.23, 1.77) | <0.001 |  | 1.46 (1.21, 1.76) | <0.001 |  | 1.40 (1.16, 1.70) | 0.001 |
|  |  | Low mucosal fibrosis | 1.00 |  |  | 1.00 |  |  | 1.00 |  |  | 1.00 |  |
|  |  | Moderate mucosal fibrosis | 1.41 (0.77, 2.60) | 0.264 |  | 1.37 (0.74, 2.52) | 0.314 |  | 1.37 (0.74, 2.53) | 0.316 |  | 1.33 (0.72, 2.46) | 0.362 |
|  |  | High mucosal fibrosis | 2.92 (1.70, 5.04) | <0.001 |  | 2.83 (1.64, 4.89) | <0.001 |  | 2.71 (1.56, 4.73) | <0.001 |  | 2.57 (1.47, 4.50) | 0.001 |
|  |  | *p*.trend |  | <0.001 |  |  | <0.001 |  |  | <0.001 |  |  | 0.001 |
|  |  | Quantified submucosal fibrosis (per SD increase) | 1.67 (1.39, 2.01) | <0.001 |  | 1.65 (1.37, 1.98) | <0.001 |  | 1.62 (1.34, 1.95) | <0.001 |  | 1.55 (1.28, 1.88) | <0.001 |
|  |  | Low submucosal fibrosis | 1.00 |  |  | 1.00 |  |  | 1.00 |  |  | 1.00 |  |
|  |  | Moderate submucosal fibrosis | 1.57 (0.85, 2.91) | 0.154 |  | 1.74 (0.93, 3.25) | 0.086 |  | 1.67 (0.88, 3.16) | 0.114 |  | 1.75 (0.92, 3.32) | 0.086 |
|  |  | High submucosal fibrosis | 3.51 (2.00, 6.16) | <0.001 |  | 3.45 (1.96, 6.06) | <0.001 |  | 3.30 (1.87, 5.81) | <0.001 |  | 2.99 (1.69, 5.32) | <0.001 |
|  |  | *p*.trend |  | <0.001 |  |  | <0.001 |  |  | <0.001 |  |  | <0.001 |
|  |  | Quantified muscularis propria fibrosis (per SD increase) | 1.30 (1.09, 1.56) | 0.004 |  | 1.29 (1.08, 1.53) | 0.005 |  | 1.26 (1.06, 1.51) | 0.011 |  | 1.20 (1.00, 1.44) | 0.048 |
|  |  | Low muscularis propria fibrosis | 1.00 |  |  | 1.00 |  |  | 1.00 |  |  | 1.00 |  |
|  |  | Moderate muscularis propria fibrosis | 0.91 (0.52, 1.60) | 0.751 |  | 0.91 (0.52, 1.60) | 0.738 |  | 0.84 (0.48, 1.49) | 0.558 |  | 0.85 (0.48, 1.51) | 0.588 |
|  |  | High muscularis propria fibrosis | 1.44 (0.87, 2.37) | 0.160 |  | 1.41 (0.85, 2.33) | 0.184 |  | 1.32 (0.80, 2.19) | 0.283 |  | 1.26 (0.76, 2.10) | 0.365 |
|  |  | *p*.trend |  | 0.148 |  |  | 0.175 |  |  | 0.361 |  |  | 0.423 |
| **Clinical recurrence** | Lesion site | Quantified mucosal fibrosis (per SD increase) | 1.73 (1.45, 2.07) | <0.001 |  | 1.73 (1.45, 2.07) | <0.001 |  | 1.71 (1.44, 2.04) | <0.001 |  | 1.47 (1.22, 1.77) | <0.001 |
|  |  | Low mucosal fibrosis | 1.00 |  |  | 1.00 |  |  | 1.00 |  |  | 1.00 |  |
|  |  | Moderate mucosal fibrosis | 1.46 (0.76, 2.82) | 0.253 |  | 1.52 (0.79, 2.92) | 0.213 |  | 1.52 (0.78, 2.95) | 0.214 |  | 1.48 (0.76, 2.88) | 0.244 |
|  |  | High mucosal fibrosis | 3.98 (2.20, 7.22) | <0.001 |  | 3.97 (2.18, 7.21) | <0.001 |  | 4.07 (2.24, 7.40) | <0.001 |  | 3.19 (1.72, 5.92) | <0.001 |
|  |  | *p*.trend |  | <0.001 |  |  | <0.001 |  |  | <0.001 |  |  | <0.001 |
|  |  | Quantified submucosal fibrosis (per SD increase) | 2.01 (1.67, 2.42) | <0.001 |  | 2.02 (1.67, 2.43) | <0.001 |  | 2.02 (1.67, 2.46) | <0.001 |  | 1.95 (1.61, 2.38) | <0.001 |
|  |  | Low submucosal fibrosis | 1.00 |  |  | 1.00 |  |  | 1.00 |  |  | 1.00 |  |
|  |  | Moderate submucosal fibrosis | 2.93 (1.52, 5.65) | 0.001 |  | 2.93 (1.52, 5.65) | 0.001 |  | 2.72 (1.40, 5.30) | 0.003 |  | 2.92 (1.46, 5.84) | 0.002 |
|  |  | High submucosal fibrosis | 6.30 (3.34, 11.86) | <0.001 |  | 6.25 (3.31, 11.80) | <0.001 |  | 5.94 (3.10, 11.40) | <0.001 |  | 6.33 (3.16, 12.65) | <0.001 |
|  |  | *p*.trend |  | <0.001 |  |  | <0.001 |  |  | <0.001 |  |  | <0.001 |
|  |  | Quantified muscularis propria fibrosis (per SD increase) | 1.90 (1.61, 2.24) | <0.001 |  | 1.97 (1.64, 2.37) | <0.001 |  | 1.93 (1.60, 2.32) | <0.001 |  | 1.65 (1.37, 1.99) | <0.001 |
|  |  | Low muscularis propria fibrosis | 1.00 |  |  | 1.00 |  |  | 1.00 |  |  | 1.00 |  |
|  |  | Moderate muscularis propria fibrosis | 2.34 (1.20, 4.55) | 0.012 |  | 2.40 (1.23, 4.67) | 0.010 |  | 2.39 (1.22, 4.67) | 0.011 |  | 2.19 (1.12, 4.30) | 0.022 |
|  |  | High muscularis propria fibrosis | 5.88 (3.16, 10.94) | <0.001 |  | 5.93 (3.18, 11.06) | <0.001 |  | 5.63 (3.00, 10.56) | <0.001 |  | 4.40 (2.31, 8.37) | <0.001 |
|  |  | *p*.trend |  | <0.001 |  |  | <0.001 |  |  | <0.001 |  |  | <0.001 |
|  | Resection margin | Quantified mucosal fibrosis (per SD increase) | 1.39 (1.16, 1.66) | <0.001 |  | 1.38 (1.16, 1.65) | <0.001 |  | 1.39 (1.16, 1.66) | <0.001 |  | 1.33 (1.10, 1.60) | 0.003 |
|  |  | Low mucosal fibrosis | 1.00 |  |  | 1.00 |  |  | 1.00 |  |  | 1.00 |  |
|  |  | Moderate mucosal fibrosis | 1.40 (0.81, 2.44) | 0.228 |  | 1.47 (0.85, 2.57) | 0.170 |  | 1.52 (0.87, 2.66) | 0.142 |  | 1.41 (0.80, 2.48) | 0.238 |
|  |  | High mucosal fibrosis | 2.18 (1.31, 3.66) | 0.003 |  | 2.26 (1.35, 3.80) | 0.002 |  | 2.17 (1.28, 3.67) | 0.004 |  | 2.04 (1.19, 3.48) | 0.009 |
|  |  | *p*.trend |  | 0.003 |  |  | 0.002 |  |  | 0.007 |  |  | 0.015 |
|  |  | Quantified submucosal fibrosis (per SD increase) | 1.50 (1.25, 1.80) | <0.001 |  | 1.50 (1.25, 1.79) | <0.001 |  | 1.51 (1.26, 1.81) | <0.001 |  | 1.41 (1.17, 1.70) | <0.001 |
|  |  | Low submucosal fibrosis | 1.00 |  |  | 1.000 |  |  | 1.00 |  |  | 1.00 |  |
|  |  | Moderate submucosal fibrosis | 0.92 (0.51, 1.64) | 0.779 |  | 0.95 (0.52, 1.71) | 0.860 |  | 0.95 (0.52, 1.73) | 0.867 |  | 1.05 (0.57, 1.92) | 0.876 |
|  |  | High submucosal fibrosis | 2.31 (1.42, 3.77) | 0.001 |  | 2.32 (1.42, 3.79) | 0.001 |  | 2.31 (1.41, 3.78) | 0.001 |  | 1.97 (1.19, 3.24) | 0.008 |
|  |  | *p*.trend |  | 0.001 |  |  | 0.001 |  |  | 0.001 |  |  | 0.008 |
|  |  | Quantified muscularis propria fibrosis (per SD increase) | 1.23 (1.03, 1.47) | 0.020 |  | 1.23 (1.04, 1.47) | 0.018 |  | 1.22 (1.02, 1.45) | 0.029 |  | 1.14 (0.95, 1.35) | 0.153 |
|  |  | Low muscularis propria fibrosis | 1.00 |  |  | 1.00 |  |  | 1.00 |  |  | 1.00 |  |
|  |  | Moderate muscularis propria fibrosis | 0.95 (0.54, 1.66) | 0.844 |  | 0.97 (0.55, 1.70) | 0.911 |  | 0.92 (0.52, 1.61) | 0.764 |  | 0.99 (0.56, 1.77) | 0.986 |
|  |  | High muscularis propria fibrosis | 1.66 (1.03, 2.69) | 0.039 |  | 1.67 (1.03, 2.70) | 0.038 |  | 1.55 (0.95, 2.52) | 0.077 |  | 1.54 (0.94, 2.51) | 0.087 |
|  |  | *p*.trend |  | 0.030 |  |  | 0.029 |  |  | 0.079 |  |  | 0.070 |

Model 0: crude;

Model 1: adjusted with crude, age, sex;

Model 2: adjusted with crude, age, sex, smoking, behavior, past surgical history;

Model 3: adjusted with crude, age, sex, smoking, behavior, past surgical history, total granuloma, total plexitis, active inflammation at surgical margin

**Table S3. Subgroup analysis of hazard ratios for endoscopic recurrence according to transmural fibrosis at lesion site**

| **Groups** | **Quantified fibrosis (per SD increase)** | |  | **Moderate fibrosis** | |  | **High fibrosis** | |
| --- | --- | --- | --- | --- | --- | --- | --- | --- |
|  | **HR^*^ (95%CI) model3** | ***P*-value** |  | **HR (95%CI) model3** | ***P*-value** |  | **HR (95%CI) model3** | ***P*-value** |
| **All patients** | 1.46 (1.18, 1.80) | 0.001 |  | 2.27 (1.17, 4.39) | 0.015 |  | 3.02 (1.61, 5.66) | 0.001 |
| **Sex** |  |  |  |  |  |  |  |  |
| male | 1.50 (1.14, 1.98) | 0.004 |  | 1.61 (0.67, 3.89) | 0.288 |  | 3.66 (1.58, 8.44) | 0.002 |
| female | 1.53 (1.04, 2.27) | 0.033 |  | 2.90 (1.00, 8.44) | 0.051 |  | 3.03 (1.07, 8.58) | 0.036 |
| **Age** |  |  |  |  |  |  |  |  |
| young | 1.62 (0.98, 2.67) | 0.058 |  | 2.12 (0.61, 7.35) | 0.236 |  | 2.64 (0.80, 8.75) | 0.112 |
| elder | 1.46 (1.14, 1.86) | 0.002 |  | 2.08 (0.93, 4.65) | 0.075 |  | 3.12 (1.46, 6.65) | 0.003 |
| **Penetration** |  |  |  |  |  |  |  |  |
| no | 1.48 (1.16, 1.90) | 0.002 |  | 1.74 (0.81, 3.75) | 0.158 |  | 3.11 (1.50, 6.42) | 0.002 |
| yes | 1.54 (0.96, 2.45) | 0.071 |  | 7.37 (1.78, 30.55) | 0.006 |  | 5.29 (1.26, 22.20) | 0.023 |

^*^:Hazard ratios (HRs) were estimated using multivariable Cox proportional hazards Model 3: adjusted with crude, age, sex, smoking, behavior, past surgical history, total granuloma, total plexitis, active inflammation at surgical margin.

For categorical fibrosis analyses, the low-fibrosis group served as the reference.

**Table S4. Subgroup analysis of hazard ratios for clinical recurrence according to transmural fibrosis at lesion site**

| **Groups** | **Quantified fibrosis (per SD increase)** | |  | **Moderate fibrosis** | |  | **High fibrosis** | |
| --- | --- | --- | --- | --- | --- | --- | --- | --- |
|  | **HR^*^ (95%CI) model3** | ***P*-value** |  | **HR (95%CI) model3** | ***P*-value** |  | **HR (95%CI) model3** | ***P*-value** |
| **All patients** | 1.95 (1.59, 2.39) | <0.001 |  | 3.62 (1.75, 7.52) | 0.001 |  | 5.91 (2.89, 12.06) | <0.001 |
| **Sex** |  |  |  |  |  |  |  |  |
| male | 1.80 (1.36, 2.38) | <0.001 |  | 4.44 (1.51, 13.08) | 0.007 |  | 6.33 (2.23, 17.96) | 0.001 |
| female | 2.51 (1.69, 3.73) | <0.001 |  | 3.36 (1.20, 9.43) | 0.021 |  | 5.87 (2.13, 16.19) | 0.001 |
| **Age** |  |  |  |  |  |  |  |  |
| young | 1.55 (0.93, 2.58) | 0.094 |  | 2.83 (0.72, 11.06) | 0.135 |  | 2.01 (0.59, 6.78) | 0.262 |
| elder | 2.06 (1.64, 2.58) | <0.001 |  | 4.28 (1.69, 10.84) | 0.002 |  | 8.86 (3.52, 22.31) | <0.001 |
| **Penetration** |  |  |  |  |  |  |  |  |
| no | 2.02 (1.60, 2.54) | <0.001 |  | 3.88 (1.52, 9.89) | 0.004 |  | 7.91 (3.21, 19.49) | <0.001 |
| yes | 1.66 (1.05, 2.62) | 0.029 |  | 5.84 (1.63, 20.94) | 0.007 |  | 4.22 (1.13, 15.68) | 0.032 |

^*^:Hazard ratios (HRs) were estimated using multivariable Cox proportional hazards Model 3: adjusted with crude, age, sex, smoking, behavior, past surgical history, total granuloma, total plexitis, active inflammation at surgical margin.

For categorical fibrosis analyses, the low-fibrosis group served as the reference.

**Table S5. Subgroup analysis of hazard ratios for endoscopic recurrence according to transmural fibrosis at resection margin**

| **Groups** | **Quantified fibrosis (per SD increase)** | |  | **Moderate fibrosis** | |  | **High fibrosis** | |
| --- | --- | --- | --- | --- | --- | --- | --- | --- |
|  | **HR^*^ (95%CI) model3** | ***P*-value** |  | **HR (95%CI) model3** | ***P*-value** |  | **HR (95%CI) model3** | ***P*-value** |
| **All patients** | 1.35 (1.12, 1.63) | 0.002 |  | 1.30 (0.69, 2.44) | 0.422 |  | 2.88 (1.63, 5.09) | <0.001 |
| **Sex** |  |  |  |  |  |  |  |  |
| male | 1.48 (1.14, 1.94) | 0.004 |  | 1.25 (0.53, 2.95) | 0.605 |  | 3.70 (1.70, 8.06) | 0.001 |
| female | 1.24 (0.91, 1.68) | 0.177 |  | 1.57 (0.58, 4.24) | 0.374 |  | 2.49 (0.98, 6.30) | 0.055 |
| **Age** |  |  |  |  |  |  |  |  |
| young | 1.17 (0.84, 1.64) | 0.355 |  | 4.78 (1.24, 18.37) | 0.023 |  | 4.53 (1.36, 15.13) | 0.014 |
| elder | 1.60 (1.25, 2.05) | <0.001 |  | 0.83 (0.38, 1.78) | 0.629 |  | 2.44 (1.24, 4.82) | 0.01 |
| **Penetration** |  |  |  |  |  |  |  |  |
| no | 1.48 (1.16, 1.88) | 0.001 |  | 1.97 (0.90, 4.31) | 0.088 |  | 3.56 (1.74, 7.28) | 0.001 |
| yes | 1.26 (0.88, 1.82) | 0.211 |  | 0.70 (0.21, 2.36) | 0.564 |  | 2.42 (0.85, 6.93) | 0.098 |

^*^:Hazard ratios (HRs) were estimated using multivariable Cox proportional hazards Model 3: adjusted with crude, age, sex, smoking, behavior, past surgical history, total granuloma, total plexitis, active inflammation at surgical margin.

For categorical fibrosis analyses, the low-fibrosis group served as the reference.

**Table S6. Subgroup analysis of hazard ratios for clinical recurrence according to transmural fibrosis at resection margin**

| **Groups** | **Quantified fibrosis (per SD increase)** | |  | **Moderate fibrosis** | |  | **High fibrosis** | |
| --- | --- | --- | --- | --- | --- | --- | --- | --- |
|  | **HR^*^ (95%CI) model3** | ***P*-value** |  | **HR (95%CI) model3** | ***P*-value** |  | **HR (95%CI) model3** | ***P*-value** |
| **All patients** | 1.29 (1.09, 1.54) | 0.003 |  | 1.30 (0.70, 2.41) | 0.400 |  | 2.85 (1.62, 5.01) | <0.001 |
| **Sex** |  |  |  |  |  |  |  |  |
| male | 1.34 (1.01, 1.77) | 0.044 |  | 1.22 (0.52, 2.87) | 0.644 |  | 2.48 (1.08, 5.69) | 0.032 |
| female | 1.26 (0.97, 1.63) | 0.088 |  | 1.58 (0.63, 3.99) | 0.329 |  | 3.52 (1.45, 8.52) | 0.005 |
| **Age** |  |  |  |  |  |  |  |  |
| young | 1.02 (0.74, 1.40) | 0.894 |  | 2.23 (0.53, 9.33) | 0.272 |  | 4.30 (1.20, 15.34) | 0.025 |
| elder | 1.46 (1.18, 1.82) | 0.001 |  | 1.06 (0.53, 2.15) | 0.862 |  | 2.68 (1.37, 5.23) | 0.004 |
| **Penetration** |  |  |  |  |  |  |  |  |
| no | 1.34 (1.08, 1.66) | 0.007 |  | 1.45 (0.67, 3.10) | 0.345 |  | 2.88 (1.44, 5.74) | 0.003 |
| yes | 1.22 (0.86, 1.74) | 0.259 |  | 1.01 (0.30, 3.39) | 0.986 |  | 2.60 (0.87, 7.76) | 0.086 |

^*^:Hazard ratios (HRs) were estimated using multivariable Cox proportional hazards Model 3: adjusted with crude, age, sex, smoking, behavior, past surgical history, total granuloma, total plexitis, active inflammation at surgical margin.

For categorical fibrosis analyses, the low-fibrosis group served as the reference.

**Table S7. The nested multifactorial Cox regression analysis for transmural fibrosis at different resection margins and post-operation recurrence**

| **Outcome** | **Transmural fibrosis** | **Model 0** | |  | **Model 1** | |  | **Model 2** | |  | **Model 3** | |
| --- | --- | --- | --- | --- | --- | --- | --- | --- | --- | --- | --- | --- |
|  |  | **HR (95%CI)** | ***P*-value** |  | **HR (95%CI)** | ***P*-value** |  | **HR (95%CI)** | ***P*-value** |  | **HR (95%CI)** | ***P*-value** |
| **Endoscopic recurrence** | Proximal margin (per SD increase) | 1.41 (1.15, 1.73) | 0.001 |  | 1.41 (1.15, 1.73) | 0.001 |  | 1.38 (1.12, 1.69) | 0.002 |  | 1.28 (1.02, 1.60) | 0.034 |
|  | Proximal margin (moderate vs low) | 0.89 (0.41, 1.91) | 0.759 |  | 0.89 (0.41, 1.91) | 0.756 |  | 0.83 (0.38, 1.81) | 0.644 |  | 0.88 (0.40, 1.93) | 0.753 |
|  | Proximal margin (high vs low) | 2.27 (1.19, 4.33) | 0.013 |  | 2.26 (1.18, 4.33) | 0.013 |  | 2.18 (1.13, 4.21) | 0.02 |  | 1.96 (1.00, 3.84) | 0.051 |
|  | *p*.trend |  | 0.002 |  |  | 0.002 |  |  | 0.004 |  |  | 0.021 |
|  | Distal margin (per SD increase) | 2.37 (1.40, 4.03) | 0.001 |  | 2.50 (1.43, 4.37) | 0.001 |  | 2.28 (1.28, 4.06) | 0.005 |  | 2.31 (1.23, 4.35) | 0.009 |
|  | Distal margin (moderate vs low) | 1.17 (0.28, 4.96) | 0.829 |  | 1.13 (0.27, 4.77) | 0.872 |  | 1.17 (0.24, 5.59) | 0.848 |  | 0.88 (0.16, 4.91) | 0.882 |
|  | Distal margin (high vs low) | 6.60 (2.02, 21.49) | 0.002 |  | 6.41 (1.97, 20.88) | 0.002 |  | 5.96 (1.69, 21.06) | 0.006 |  | 6.01 (1.63, 22.17) | 0.007 |
|  | *p*.trend |  | 0.001 |  |  | 0.001 |  |  | 0.003 |  |  | 0.004 |
| **Clinical recurrence** | Proximal margin (per SD increase) | 1.41 (1.17, 1.70) | <0.001 |  | 1.41 (1.17, 1.69) | <0.001 |  | 1.41 (1.17, 1.70) | <0.001 |  | 1.24 (1.02, 1.51) | 0.033 |
|  | Proximal margin (moderate vs low) | 0.73 (0.33, 1.64) | 0.452 |  | 0.71 (0.32, 1.59) | 0.402 |  | 0.71 (0.31, 1.60) | 0.409 |  | 0.78 (0.34, 1.77) | 0.551 |
|  | Proximal margin (high vs low) | 2.47 (1.31, 4.64) | 0.005 |  | 2.46 (1.31, 4.62) | 0.005 |  | 2.58 (1.36, 4.91) | 0.004 |  | 2.28 (1.18, 4.42) | 0.015 |
|  | *p*.trend |  | <0.001 |  |  | <0.001 |  |  | <0.001 |  |  | 0.002 |
|  | Distal margin (per SD increase) | 2.33 (1.34, 4.05) | 0.003 |  | 2.51 (1.38, 4.57) | 0.003 |  | 3.01 (1.65, 5.50) | <0.001 |  | 2.49 (1.34, 4.62) | 0.004 |
|  | Distal margin (moderate vs low) | 4.03 (1.17, 13.91) | 0.027 |  | 3.85 (1.12, 13.29) | 0.033 |  | 6.36 (1.50, 26.87) | 0.012 |  | 3.21 (0.69, 14.80) | 0.136 |
|  | Distal margin (high vs low) | 8.22 (2.35, 28.82) | 0.001 |  | 7.39 (2.10, 25.96) | 0.002 |  | 18.01 (3.73, 86.83) | <0.001 |  | 18.62 (3.42, 101.43) | 0.001 |
|  | *p*.trend |  | 0.001 |  |  | 0.001 |  |  | <0.001 |  |  | 0.001 |

**Table S8. Univariable Cox regression for post-operation endoscopic and clinical recurrence**

| **Characteristics** | **Endoscopic recurrence** | |  | **Clinical recurrence** | |
| --- | --- | --- | --- | --- | --- |
|  | **HR (95%CI)** | ***P*-value** |  | **HR (95%CI)** | ***P*-value** |
| Age | 0.98 (0.97, 1.00) | 0.063 |  | 1.01 (0.99, 1.02) | 0.388 |
| Female | 1.19 (0.77, 1.84) | 0.441 |  | 1.59 (1.05, 2.40) | 0.028 |
| BMI | 0.97 (0.90, 1.04) | 0.372 |  | 0.95 (0.89, 1.02) | 0.182 |
| Age at diagnosis group |  |  |  |  |  |
| A2 | 1.00 |  |  | 1.00 |  |
| A1 | 1.55 (0.56, 4.29) | 0.397 |  | 0.72 (0.17, 2.95) | 0.646 |
| A3 | 0.87 (0.54, 1.38) | 0.550 |  | 1.34 (0.88, 2.05) | 0.169 |
| Location |  |  |  |  |  |
| L1 | 1.00 |  |  | 1.00 |  |
| L3 | 1.80 (1.17, 2.76) | 0.008 |  | 1.07 (0.71, 1.62) | 0.751 |
| L4 | 1.37 (0.55, 3.38) | 0.501 |  | 1.28 (0.52, 3.16) | 0.594 |
| Penetrating behavior | 0.73 (0.45, 1.19) | 0.209 |  | 0.73 (0.46, 1.15) | 0.176 |
| Perianal involvement | 1.26 (0.74, 2.14) | 0.402 |  | 1.42 (0.85, 2.37) | 0.177 |
| Smoking | 0.23 (0.06, 0.94) | 0.040 |  | 0.42 (0.15, 1.14) | 0.087 |
| Intra abdominal abscess | 0.99 (0.47, 2.05) | 0.973 |  | 0.48 (0.19, 1.19) | 0.114 |
| Past surgical history | 1.01 (0.65, 1.58) | 0.968 |  | 1.37 (0.90, 2.08) | 0.138 |
| Ileocecectomy | 1.58 (1.02, 2.42) | 0.039 |  | 0.73 (0.47, 1.13) | 0.159 |
| Enterostomy | 0.83 (0.44, 1.58) | 0.574 |  | 0.71 (0.38, 1.33) | 0.287 |
| SSA usage | 1.07 (0.64, 1.80) | 0.787 |  | 1.22 (0.74, 2.02) | 0.441 |
| Albumin level |  |  |  |  |  |
| Normal | 1.00 |  |  | 1.00 |  |
| Mild decrease | 1.10 (0.63, 1.92) | 0.746 |  | 1.30 (0.78, 2.17) | 0.321 |
| Moderate decrease | 1.42 (0.78, 2.58) | 0.245 |  | 1.19 (0.65, 2.16) | 0.571 |
| Severe decrease | 0.72 (0.10, 5.23) | 0.747 |  | 1.28 (0.31, 5.25) | 0.733 |
| CRP elevated | 1.03 (0.62, 1.72) | 0.912 |  | 1.45 (0.91, 2.33) | 0.121 |
| Pre surgery medication |  |  |  |  |  |
| No medication | 1.00 |  |  | 1.00 |  |
| 5 ASA | 1.14 (0.74, 1.75) | 0.554 |  | 0.95 (0.63, 1.44) | 0.824 |
| Pre surgery steroid | 1.39 (0.87, 2.22) | 0.172 |  | 1.44 (0.92, 2.25) | 0.11 |
| Pre surgery immune | 1.09 (0.70, 1.70) | 0.693 |  | 1.54 (1.02, 2.33) | 0.042 |
| Pre surgery biologics | 1.64 (1.00, 2.68) | 0.050 |  | 1.80 (1.12, 2.90) | 0.015 |
| Post surgery medication | 2.07 (0.95, 4.52) | 0.066 |  | 1.31 (0.71, 2.41) | 0.392 |
| Granuloma at lesion | 3.74 (2.22, 6.30) | <0.001 |  | 7.37 (3.82, 14.22) | <0.001 |
| Granuloma at resection margin | 1.71 (1.10, 2.66) | 0.016 |  | 3.08 (2.04, 4.66) | <0.001 |
| Plexitis at lesion | 2.91 (1.34, 6.31) | 0.007 |  | 10.86 (2.67, 44.11) | 0.001 |
| Plexitis at resection margin | 1.67 (1.08, 2.57) | 0.020 |  | 2.63 (1.69, 4.11) | <0.001 |
| Active inflammation at resection margin | 1.23 (0.75, 2.01) | 0.416 |  | 1.13 (0.71, 1.82) | 0.602 |

**Table S9. Adjusted hazard ratios for endoscopic and clinical recurrence according to transmural fibrosis in sensitive analyses**

| **Site** | **Fibrosis** | **Endoscopic recurrence** | |  | **Clinical recurrence** | |
| --- | --- | --- | --- | --- | --- | --- |
|  |  | **HR (95%CI)** | ***P*-value** |  | **HR (95%CI)** | ***P*-value** |
| **Lesion site** | Quantified transmural fibrosis (per SD increase) | 1.63 (1.29, 2.05) | <0.001 |  | 2.21 (1.78, 2.75) | <0.001 |
|  | Low transmural fibrosis | 1.00 |  |  | 1.00 |  |
|  | Moderate transmural fibrosis | 2.20 (1.13, 4.28) | 0.021 |  | 2.97 (1.43, 6.17) | 0.003 |
|  | High transmural fibrosis | 3.44 (1.80, 6.58) | <0.001 |  | 5.64 (2.76, 11.52) | <0.001 |
| **Resection margin** | Quantified transmural fibrosis (per SD increase) | 1.50 (1.25, 1.80) | <0.001 |  | 1.51 (1.28, 1.77) | <0.001 |
|  | Low transmural fibrosis | 1.00 |  |  | 1.00 |  |
|  | Moderate transmural fibrosis | 1.40 (0.73, 2.70) | 0.311 |  | 1.56 (0.84, 2.88) | 0.155 |
|  | High transmural fibrosis | 3.84 (2.14, 6.91) | <0.001 |  | 3.88 (2.23, 6.76) | <0.001 |

**Fig S1**


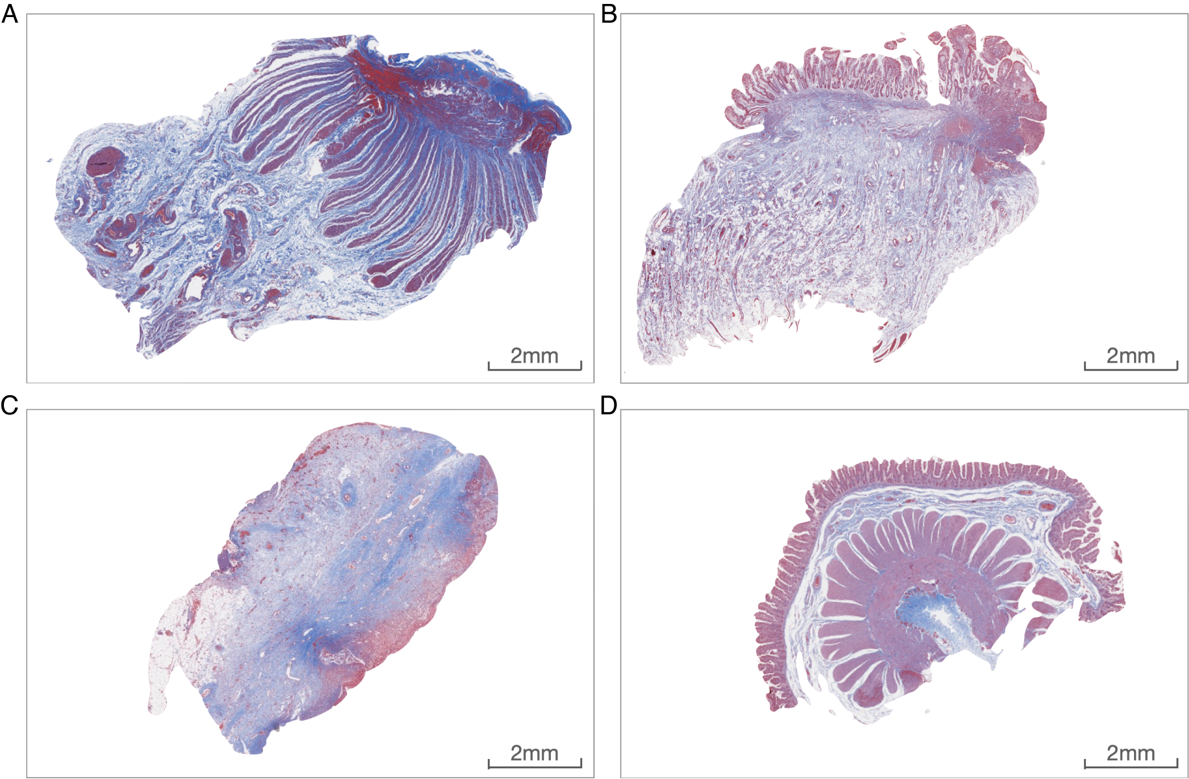


**Fig S1. Quality control criteria for tissue section selection.**

(A) Section excluded due to absence of the mucosa layer.

(B) Section excluded due to absence of the muscularis propria.

(C) Section excluded due to inadequate representation of both mucosa and muscularis propria.

(D) A high-quality section meeting all inclusion criteria, with intact mucosal, submucosal, and muscular layers, which was retained for final analysis. A scale bar of 2 mm was provided for all panels.

**Fig S2**


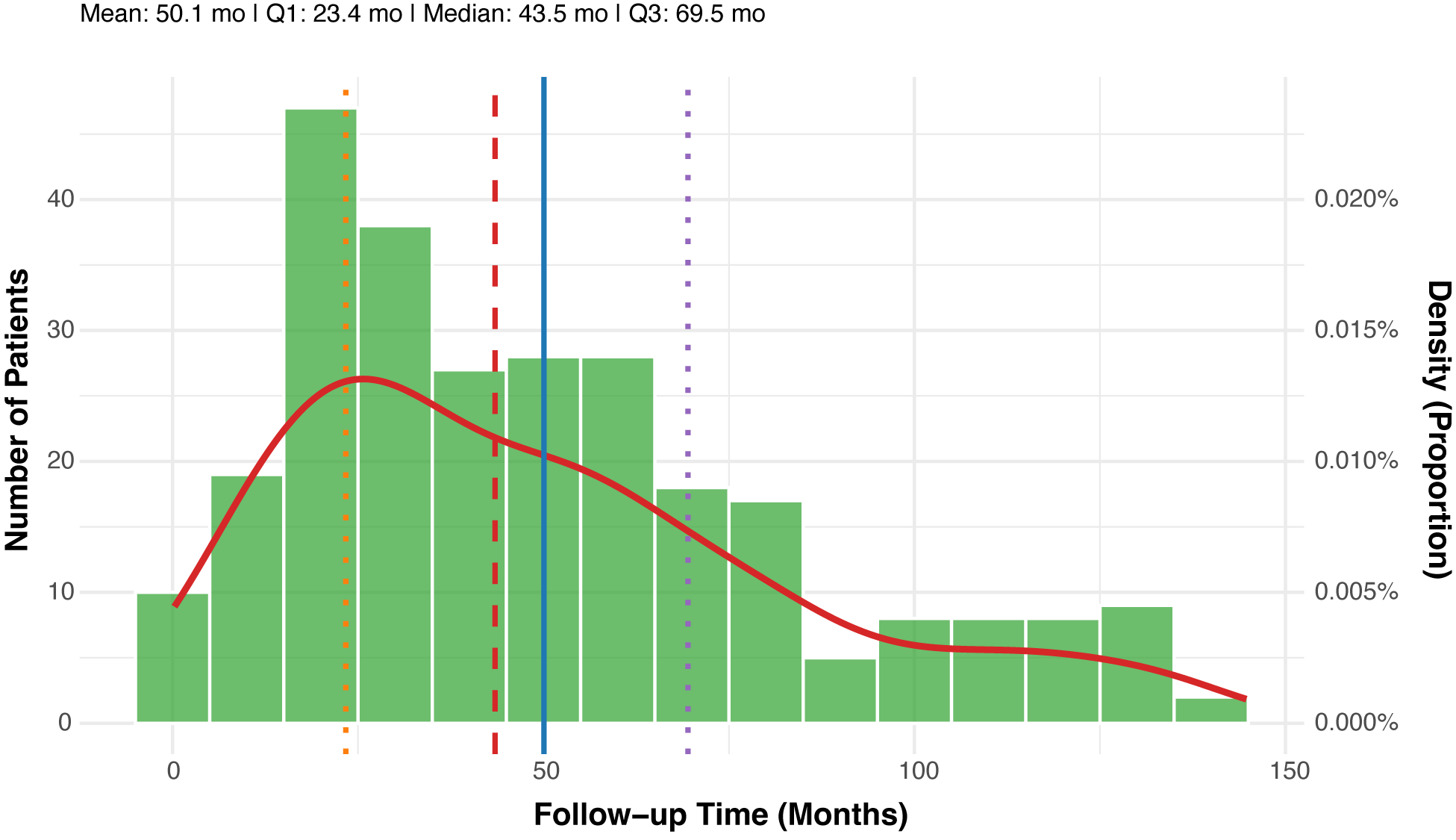


**Fig S2. Postoperative follow-up duration of the study cohort.**

The distribution of postoperative follow-up time for all patients. The median follow-up was 43.5 months (interquartile range, 23.4-69.5 months); the mean follow-up was 50.1 months. The histogram illustrates the distribution of patient counts, with the corresponding density distribution shown by the red curve. The mean, median, first quartile (Q1), and third quartile (Q3) are indicated by a blue solid line, a red dashed line, a yellow dashed line, and a purple dashed line, respectively.

**Fig S3**

**
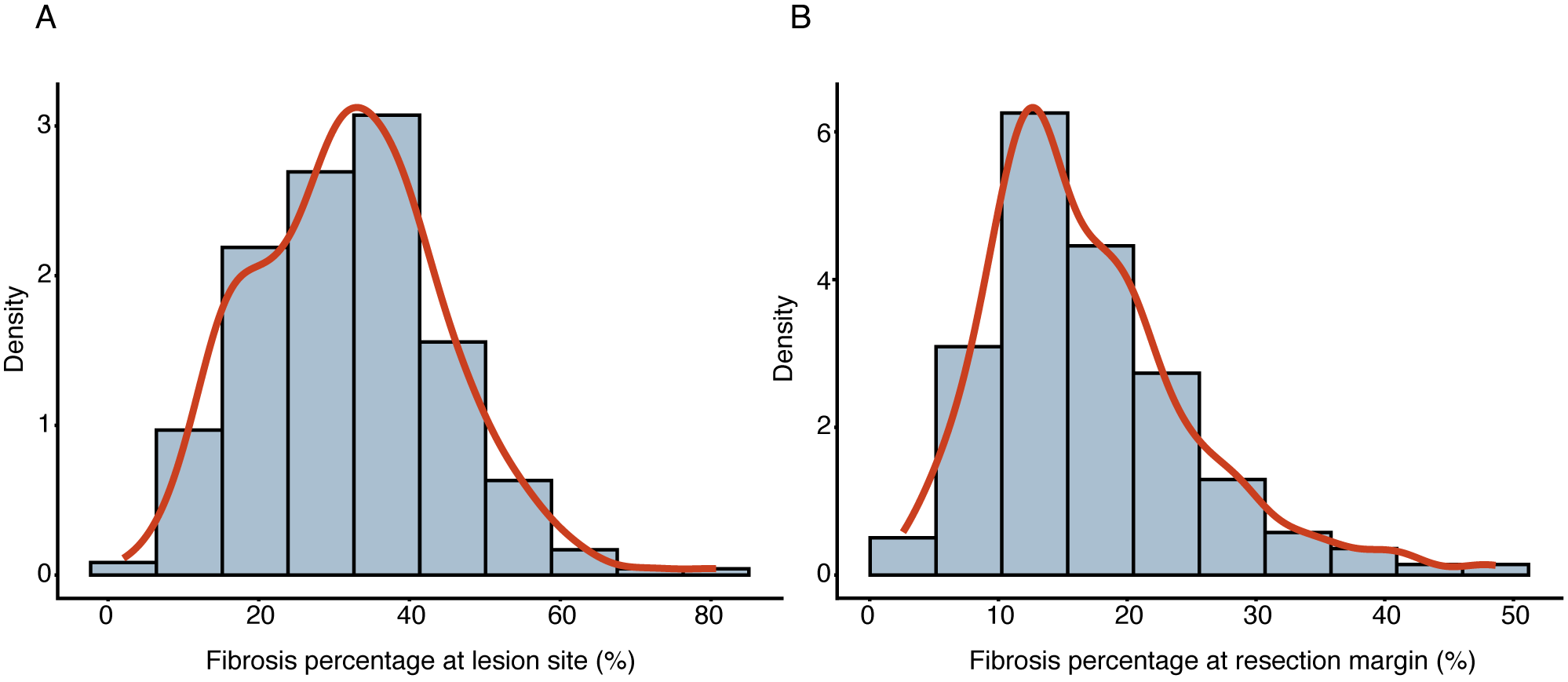
**

**Fig S3. The distribution of transmural fibrosis percentage across all cases.**

1. Histogram with density curve showing the transmural fibrosis distribution at lesion site
2. Histogram with density curve showing the transmural fibrosis distribution at resection margin

The horizontal axis in both panels represents the transmural fibrosis proportion. The fibrosis percentage is calculated as the fibrotic area divided by the total tissue area. The vertical axis in both panels shows the density, which represents a smoothed estimate of the relative frequency. The overlaid kernel density curve illustrates the continuous distribution of the data.

**Fig S4**


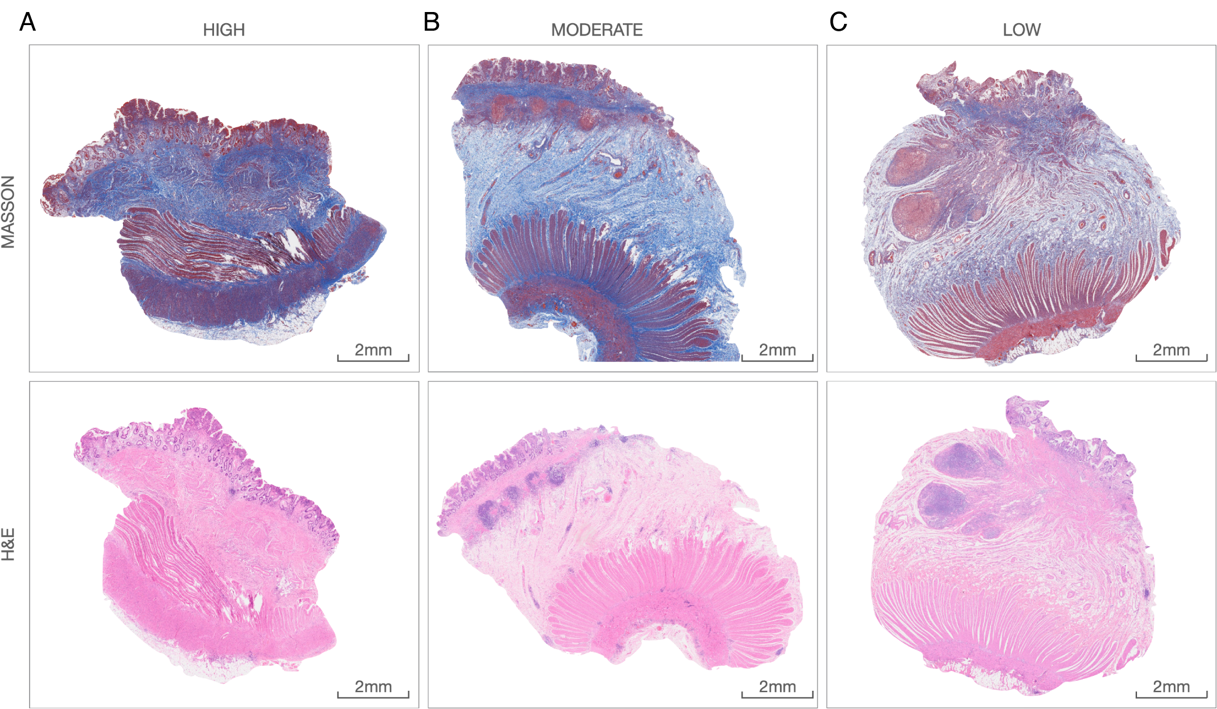


**Fig S4. Representative histopathological images for each fibrosis grade.**

The cohort was divided into tertiles (low, moderate, high) based on the ranked distribution of the fibrotic area proportion.

(A) Representative images of high-grade fibrosis.

(B) Representative images of moderate-grade fibrosis.

(C) Representative images of low-grade fibrosis.

For each grade (A–C), the top panel shows Masson’s trichrome staining (collagen in blue), and the bottom panel shows the corresponding hematoxylin and eosin (H&E) staining. Scale bar, 2 mm (applies to all panels).

**Fig S5**


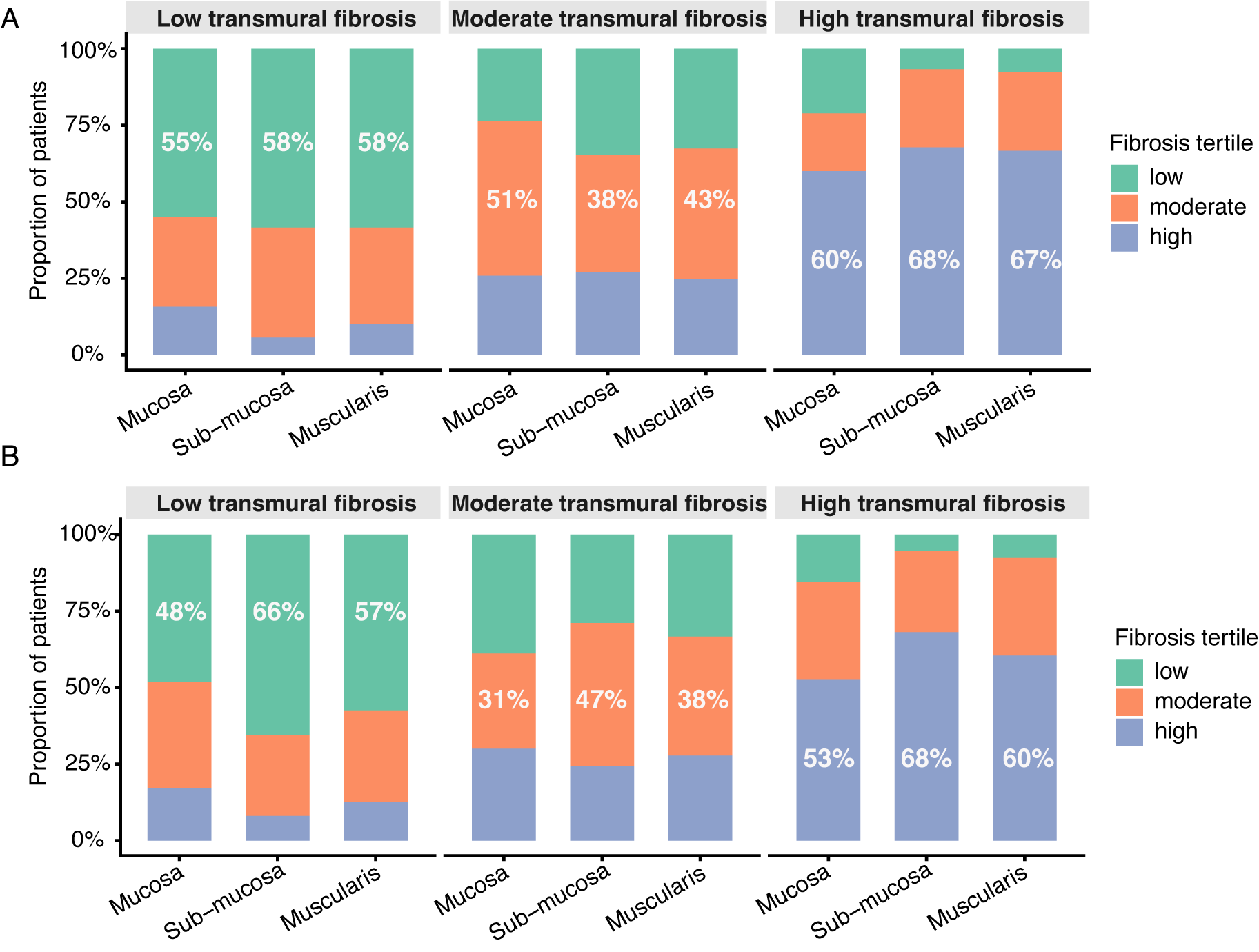


**Fig S5. Stratification of transmural and layer-specific intestinal fibrosis.**

The histogram illustrates the stratification of intestinal fibrosis severity. The cohort was first divided into tertiles (Low, Moderate, High) based on the ranked distribution of the overall transmural fibrotic area proportion. Within each tertile group, the relative distribution of fibrosis severity across the mucosa, submucosa, and muscularis propria was displayed. The analysis was performed separately for (A) the primary lesion site and (B) the resection margin.

**Reference**

[1] LI X H, FANG Z N, GUAN T M, et al. A novel collagen area fraction index to quantitatively assess bowel fibrosis in patients with Crohn's disease [J]. BMC Gastroenterol, 2019, 19(1): 180.
